# Microstructured Liquid Metal‐Based Embedded‐Type Sensor Array for Curved Pressure Mapping

**DOI:** 10.1002/advs.202413233

**Published:** 2024-11-25

**Authors:** Haoyu Li, Chengjun Zhang, Hongyu Xu, Qing Yang, Zexiang Luo, Cheng Li, Lin Kai, Yizhao Meng, Jialiang Zhang, Jie Liang, Feng Chen

**Affiliations:** ^1^ State Key Laboratory for Manufacturing System Engineering and Shaanxi Key Laboratory of Photonics Technology for Information School of Electronic Science and Engineering Xi'an Jiaotong University Xi'an 710049 P. R. China; ^2^ School of Instrument Science and Technology Xi'an Jiaotong University Xi'an 710049 P. R. China

**Keywords:** bio‐inspired structure, femtosecond laser, liquid metal, objects recognition, pressure sensor array

## Abstract

Human hands can envelop the surface of an object and recognize its shape through touch. However, existing stretchable haptic sensors exhibit limited flexibility and stability to detect pressure during deformation, while also solely achieving recognition of planar objects. Inspired by the structure of skin tissue, an embedded construction‐enabled liquid metal‐based e‐skin composed of a liquid metal microstructured electrode (LM‐ME) array is fabricated for curved pressure mapping. The embedded LM‐ME‐based sensor elements are fabricated by using femtosecond laser‐induced micro/nanostructures and water/hydrogel assisted patterning method, which enables high sensitivity (7.42 kPa^−1^ in the range of 0–0.1 kPa) and high stability through an interlinked support isolation structure for the sensor units. The sensor array with a high interfacial toughness of 1328 J m^−2^ can maintain pressure sensation under bending and stretching conditions. Additionally, the embedded construction and laser‐induced bumps effectively reduce crosstalk from 58 to 7.8% compared to conventional flexible sensors with shared surfaces. The stretchable and mechanically stable sensor arrays possess shape‐adaptability that enables pressure mapping on non‐flat surfaces, which has great potential for object recognition in robotic skins and human‐computer interaction.

## Graphical abstract

An all‐soft pressure sensor array is presented by femtosecond laser‐induced micro/nanostructures and developing the formation method of liquid metal film on a rough surface of PDMS. The sensor array exhibits stability, high sensitivity, and low cross‐talk by optimizing the structure design, enabling conformal and shape‐adaptable attachment to curved surfaces for object recognition.

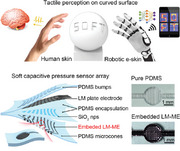

## 1 Introduction

Sense of touch serves as the primary means for individuals to gain an understanding of the world and establish communication with the external environment. The tactile sensation of the human hand depends on a variety of mechanoreceptors in the dermal‐epidermal layer of the skin, as well as spinosum, which plays a key role in enhancing mechanical perception.^[^ ^1^ ^]^ Meanwhile, the receptors are embedded in the skin tissue and grow together with surrounding tissue, forming robust and tough interfaces.^[^ ^2^ ^]^ Such firm interlinks between the layers allow the skin to operate normally in a variety of deformations such as stretching, torsion, bending, and showing high spatial resolution.^[^ ^2^, ^3^ ^]^ These intricate networks of nerves and sensory receptors in our hands allow us to accurately perceive and interact with objects effortlessly such as grasping objects, playing a musical instrument, and writing with a pen. Inspired by nature's design, scientists have developed flexible pressure sensors or electronic skins (e‐skins) that simulate the ability of human skin to sense external environmental stimuli.^[^ ^4^ ^]^ These e‐skins are capable of converting external physical stimuli into electrical signals while maintaining the same flexibility and stretchability as natural human skin. In contrast to conventional electronic devices based on rigid materials and networks, e‐skin has great potential in robotic tactile perception, wearable electronic devices, and intelligent medical treatment.^[^ ^5^ ^]^ At present, considerable efforts have been dedicated to developing innovative materials for producing highly performing electron devices, such as flexible pressure sensors mainly prepared by ITO, carbon nanotubes, metal (Au, Ag, and Cu) nanowires, conductive polymers, etc.^[^ ^6^ ^]^ However, these electrode materials exhibit limitations in terms of flexibility or conductivity, lack compatibility with soft skin and tissue, and fail to function properly under deformation.

In recent years, gallium‐based liquid metals (LM) have attracted considerable attention due to their excellent electrical/thermal conductivity, low vapor pressure, inherent fluidity and stretchability, and good biocompatibility.^[^ ^7^ ^]^ LM has shown huge potential in various electronic skin components, such as stretchable electrodes/circuits, flexible interconnections, flexible actuators, flexible on‐off devices, etc.^[^ ^8^ ^]^ However, for a significant category of components as flexible mechanical sensors, there is a need for improvement in the performance of LM‐based devices. The capacitive pressure sensor is widely acclaimed for its simplistic design, exceptional stability, low power consumption, and temperature independence. The introduction of microstructures has been proven to effectively enhance the sensitivity of pressure sensors.^[^ ^9^ ^]^ Specifically, the preparation of micro‐structured electrodes can significantly improve their pressure sensitivity.^[^ ^6^, ^10^ ^]^ However, since LM is an amorphous liquid phase, and due to their large surface tension and the presence of an external gallium oxide encasing, it is difficult to print the LM on surfaces with micro‐ or nanoscale structures.^[^ ^11^ ^]^ The printing of LM on the surface of materials is essentially an issue of surface wettability. Most researchers have used metalphilic Cu and Au as an intermediate layer to improve the surface affinity of LM and substrate.^[^ ^12^ ^]^ However, the rigid metal affects the intrinsic tensile properties of LM and greatly limits the stretchability of the sensor. Therefore, it is necessary to break through the limitation of LM wettability on structured elasticity substrate and improve the sensing performance of pressure sensors based on LM.

To meet more sophisticated application scenarios, the e‐skin should possess not only high‐pressure sensitivity but also stability of sensor array withstanding various deformations,^[^ ^1^, ^13^ ^]^ while minimizing crosstalk between pixels to ensure precise measurements. For instance, in robotics or prosthetics applications where the e‐skin needs to conform to irregular surfaces or undergo stretching and bending motions.^[^ ^14^ ^]^ Existing electronic skin or flexible pressure sensors typically exhibit multi‐layer device configurations. However, the absence of interface bonding results in inadequate signal stability and susceptibility to separation during bending, torsion, and stretching. Consequently, this leads to the relative displacement of pixel points and subsequent signal distortion. As a result, these devices face challenges in functioning effectively under complex mechanical deformations, such as the operation of robots to identify curved surface patterns (requiring sensor deformation to conformal contact on the surface). Furthermore, minimizing crosstalk between pixels becomes increasingly crucial when dealing with intricate patterns or fine details on a surface since unwanted interference between neighboring pixels can lead to inaccurate measurements or misinterpretation of tactile information.^[^ ^4^, ^15^ ^]^ Normally, signal crosstalk is expected to occur between the sensing units due to stress dispersion caused by their shared plane. When external pressure is applied to a pixel, its deformation inevitably propagates to the surrounding area, thereby eliciting responses from adjacent pixels. Although system calibration is typically employed for mitigating undesired signals, it necessitates complex circuitry and additional energy consumption. Henceforth, developing techniques or designs that minimize these phenomena will greatly contribute toward achieving precise measurements in complex application scenarios.

Here, we present a stretchable and shape‐adaptative pressure sensor array composed of LM‐ME and embedded sensor units that exhibit high sensitivity, low cross‐talk, and stability under various mechanical deformation, enabling precise pressure mapping on curved surfaces. The LM‐ME array was fabricated by femtosecond (fs) laser‐induced micro/nanostructures and water/hydrogel assisted patterning method. This overcame the difficulty of printing the LM on a micro‐nano structure, thus enhancing the overall performance of the capacitive pressure sensor. The sensor exhibits high sensitivity up to 7.42 kPa^−1^, along with a low limit of detection (LOD) of 0.84 Pa and a rapid response of 54 ms. Moreover, the design of a sensor array with a support layer between embedded sensing elements improves the interface toughness of functional layers, while incorporating surface bumps to achieve pixel isolation. Consequently, the pressure sensor array exhibits exceptional stability under mechanical deformation and low cross‐talk to 7.8%, thereby enabling accurate pressure mapping on curved surfaces resembling human hands. By leveraging combined machine learning techniques, our soft pressure sensor array can be used for pressure mapping of object recognition on curved surfaces with high accuracy (99.7%) that have great application potential in the field of robotic e‐skin and wearable electronic devices.

## 2 Results and Discussion

### 2.1 Design Concept and Characteristics of Soft Pressure Sensor Array

**Figure 1:**
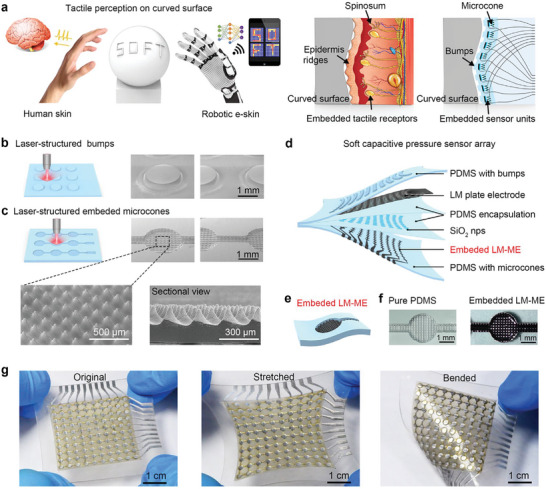
Design of human skin‐inspired soft haptic interfaces. a) Analogous to finger skin spinosum structure and embedded tactile receptors, the all‐soft LM‐based e‐skin can percept patterns on a curved surface. b,c) Schematic of laser processing of the upper layer with bumps and lower layer with embedded microcones, and the corresponding SEM figures. d) Exploded‐view schematic illustration of flexible capacitive pressure sensor array. e) Schematic of embedded LM‐ME that is the key component of e‐skin. f) Optical images of original PDMS and embedded LM‐ME. g) Digital photos of the pressure sensor array in its original, stretched, and bent state.

A pressure sensor array or tactile e‐skin inspired by human skin has been developed to simulate tactile perception and recognize objects. These pressure sensors aim to create more realistic and immersive experiences in various fields such as virtual reality, robotics, and medical applications. The perception ability of finger skin under complex conditions is attributed to four types of mechanoreceptors sensors within it, and each type has its unique structure and function (**Figure** **1**a). For example, Merkel discs (MD) are responsible for detecting touch and pressure. These receptors are embedded in the dermis of skin tissue and grow together to have tough interfaces.^[^ ^1^, ^16^ ^]^ The spinosum layer shows a microcone‐like structure densely distributed across the epidermis and the dermis and collaborates with the MD to enhance perception of external pressures and transmit signals to the nervous system. Due to unique mechanisms and structures, human skin achieves highly sensitive and accurate tactile function that enables us to perform tasks such as grasping objects and perceiving patterns on curvy surfaces flexibly. We have designed flexible sensors that replicate its elasticity, texture, sensitivity, and ability to map pressure by imitating the unique properties of human skin. Our pressure sensor array adopts a similar strategy to achieve embedded sensing elements, which is to strengthen interfacial adhesion by introducing an interlinked support layer, thereby preparing the tactile e‐skin with robust mechanical stability and low crosstalk for pressure perception on curved surfaces. Therefore, the tactile exhibits shape‐adaptability for conforming to various shapes and contours of objects, thereby enabling sense on dynamic or non‐flat surfaces to perceive shape information.

Fs laser is an ultra‐short pulse laser with the ability of high precision processing, and can directly process integrated micro or micro‐nano hierarchical rough structures on the surface of elastomer materials in an air environment.^[^ ^17^ ^]^ As shown in the Figure 1b,c, the schematic shows the process of fs laser preparing mesh patterns, including the fabricating of surface bumps on the top layer and the fabricating of embedded microcones on the bottom layer. The corresponding surface topography is shown in the inset SEM images. As for the top layer, the bumps on the surface were fabricated by laser typical line‐by‐line scanning. The interval of the adjacent distance (AD) of laser scanning lines was set at 30 µm while adopting a galvanometer machining system. The embedded microcones of the bottom layer were fabricated by orthogonally crossed line‐by‐line ablation (AD: 180 µm). The specific formation process is shown in Figure S1 (Supporting Information). By adding a pattern mask to the fs laser and combining the above flexible processing methods, microcones can be obtained in the lower layer, and a circular bumps array can be formed on the surface of the upper layer to separate the sensing unit. Insets show corresponding SEM images of a 30° view of bumps and embedded microcone. The height of the microcone can be adjusted by controlling the cycles of laser scanning and the corresponding SEM images are shown in Figure S2 (Supporting Information). Figure 1d shows an exploded view of the flexible capacitance‐type pressure sensor array sheet with 10 × 10 pixels, which is made of fully soft substrates. Polydimethylsiloxane (PDMS) is used as the substrate of the top and bottom electrode; LM is used as conductive interconnections; thin PDMS film is used as encapsulation; SiO_2_ nanoparticles (SiO_2_ nps) is used to prevent adhesion of two thin PDMS film. The thin PDMS films and SiO_2_ nps together form the dielectric layer of the capacitor. The preparation details and assembly process are shown in the Experimental Section. A key advantage of our pressure sensor array is the well‐designed embedded LM microstructured electrode (LM‐ME) in the bottom layer (Figure 1e) which significantly contributes to the superior performance of the sensor array. Figure 1f shows the optical images of pure PDMS with embedded microcone and the embedded LM‐ME which is fabricated by coating LM on embedded PDMS microcone. These designs enhance the sensitivity of pressure sensing while improving mechanical stability and effectively mitigating signal crosstalk of the sensor array, which are common issues in flexible capacitive pressure sensor arrays. Digital photos of the sensor array and under stretching and bending state are shown in Figure 1g, which indicates the sensor array has good flexibility and stretchability.

### 2.2 Preparation and Principle of Microstructured LM Electrode

**Figure 2:**
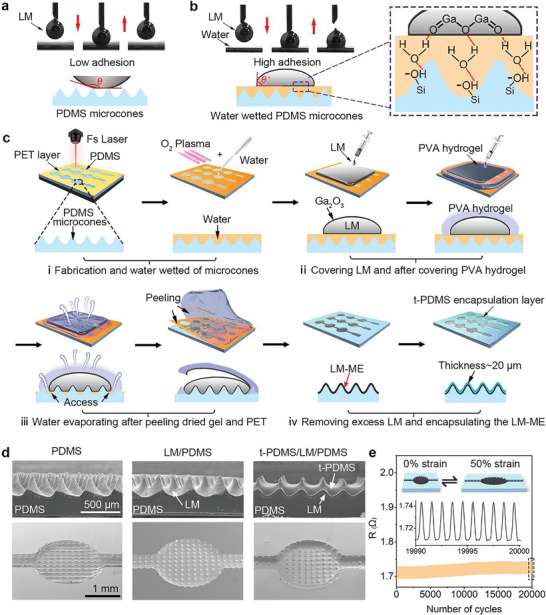
Fabrication and characterization of embedded LM‐ME. a,b) Dynamic wetting behavior of LM droplet on the original PDMS surface, fs laser‐structured PDMS microcone surface, and the water‐wetted laser‐structured PDMS surface, respectively. Insert shows the principle of high adhesion of LM on water‐wetted laser‐structured PDMS surface. c) Schematic illustration of the water/hydrogel assisted patterning method of LM onto micro‐nano structures for preparation of LM‐ME. d) SEM images of the cross‐sectional and 30° tilt‐view of three parts of LM‐ME. e) Resistance stability of LM‐ME under 50% strain cycle for 20,000 cycles.

The pattering of LM on a PDMS substrate with smooth surface is feasible and straightforward, whereas it poses a considerable challenge to form LM thin film on rough micro‐nanostructured surfaces due to the ultra‐high surface tension (800 mN m^−1^) and external oxidation layer (Ga_2_O_3_). According to Cassie's contact state,^[^ ^18^ ^]^ LM is supported on the micro‐nano structure of laser‐structured PDMS and cannot wet the surface.^[^ ^11^, ^19^ ^]^ As shown in **Figure** **2**a, the dynamic wettability of the droplet on the original PDMS surface, laser‐structured PDMS surface, and the water‐wetted laser‐structured PDMS surface were investigated respectively. An LM droplet (5 µL) extruded from the micro‐syringe contacts the surface of the PDMS substrate and then rises. The LM droplets adhered tightly to the original smooth surface of PDMS. In contrast, the laser‐structured surface completely repels the LM droplet, showing ultra‐low adhesion to LM. The LM exhibits low adhesion even changing the chemical composition of the surface and introducing a hydrophilic component to the rough surface with plasma (Figure S3, Supporting Information). This is because an air cushion in the rough structure blocks the contact between the solid‐like oxide layer of the LM and the peak of the microstructures. However, the water‐wetted rough surfaces displayed a high adhesion to LM.^[^ ^20^ ^]^ In Figure 2b, when the micro‐syringe rose, the liquid metal stuck on the surface, showing a strong adhesion on the water‐wetted microstructures. The water increases the philicity of the surface to the LM droplet, and the demonstration of the intermolecular interaction is shown in the insert mechanism diagram. After being treated with oxygen plasma, the PDMS microcones undergo a substitution reaction in which the ─CH group connected to the Si atom on the PDMS surface is replaced by ─OH, resulting in a super hydrophilicity surface. The ─OH forms a hydrogen bond with the hydrogen of the water molecule, and water molecules form a hydrogen bond with the oxygen atom of the Ga_2_O_3_ shell of LM, which produces a strong bond between the LM and substrate. As shown in Figure S4 (Supporting Information), LM droplet with larger volumes (0.1 mL) also exhibits the same property that has high adhesion on water‐wetted rough surfaces which is the key to print successful.^[^ ^21^ ^]^ Hence, the introduction and subsequent removal of water become necessary and crucial steps to achieve effective adhesion between LM and rough surfaces that are difficult to retain.

To print LM on a laser‐structured PDMS surface and fabricate LM‐ME, we employed water as an intermediate layer to enhance the adhesion between the LM and the microstructured surface, followed by introducing hydrogel for evaporation of the intermediate water. The LM is absorbed on the surface of the microstructure by a negative pressure during this process (Figure 2c). A water/hydrogel‐assisted patterning method is utilized in this process, wherein a vacuum is created to facilitate the adsorption of LM onto the microstructure surface. The detailed processes are as follows: i) the laser‐structured microcones surface is treated with oxygen plasma to transform superhydrophobicity to superhydrophilicity and is wetted by water as an intermediate medium for subsequent procedures. ii) LM and PVA hydrogel are covered on the surface in sequence. iii) As the intermediate water evaporates, the LM is adsorbed and extruded on the surface of PDMS microcones by negative pressure. Subsequently, the dried hydrogel and PET sacrificial layer were peeled off and the LM was successfully coated onto micro‐nanostructured PDMS. A necessary step in this process is pre‐reserving the contact access between the hydrogel and water, ensuring a sufficient pathway for water to evaporate through the hydrogel. iv) Finally, remove excess LM and encapsulate the micro/nano‐structured LM by a thin PDMS film (≈20 µm) to form the embedded LM‐ME. The thin PMDS film acts as the dielectric layer of the sensor and as an isolation layer that prevents the LM electrode from continuing to oxidize when exposed to air during long‐term work. Compared with other reported formation methods, this method only uses an elastic PDMS substrate and highly flexible LM to form the electrode, ensuring excellent flexibility of the pressure sensor. Figure 2d shows the SEM images of the laser‐structured PDMS, LM‐ME, and PDMS‐encapsulated LM‐ME, respectively. Enabled by the high‐precision processing and superior controllability, the microcone arrays are ablated by fs laser on the surface of the PDMS substrate. The LM completely covered the microcone and formed a thin film that could be used as flexible microstructured electrodes for pressure sensors to improve sensitivity. As the height increases, the LM cannot completely cover the tip of the microcone and cannot form a complete microstructured electrode. Therefore, an optimal sensor design with a maximum limit of 200 µm is adopted for the height of the microcone (Figure S5, Supporting Information). Figure S6 (Supporting Information) shows 100 units of as‐prepared embedded LM‐ME with stretching behavior. The resistance variation of the LM‐ME during the stretching test was depicted in Figure 2e and the resistance only slightly increased by 4% after 20,000 cycles. The result indicates that the prepared LM‐ME has good stability under strain conditions. Besides, there was no significant alteration observed in resistance during both reversing (α = 40°) and bending (r = 5 mm) in Figure S7 (Supporting Information). It is demonstrated that the prepared microstructured LM electrode possesses good flexibility and deformation stability, which has great application potential in the field of flexible electronic devices.

### 2.3 Structure and Sensing Properties of the Pressure Sensor

**Figure 3:**
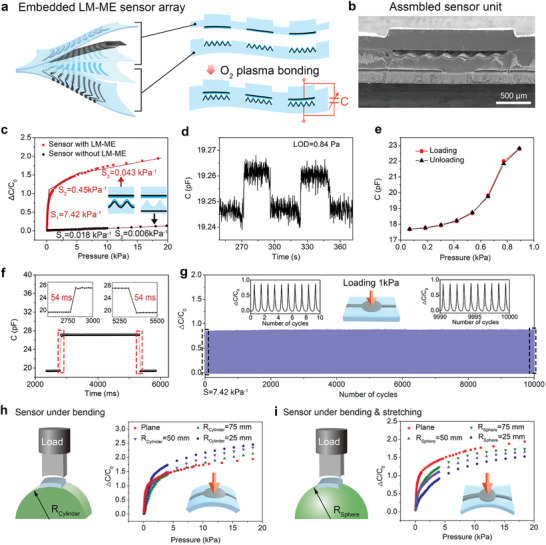
Structure and sensing properties of the all‐soft LM‐ME pressure sensor. a) Schematic diagram of the structure of the embedded LM‐ME sensor array. b) SEM images of the cross‐sectional view of the assembled sensor unit. c) Normalized change in capacitance as a function of pressure for LM‐ME sensor and conventional sensor without LM‐ME. d) Limit of detection (LOD). e) Consecutive loading and unloading process for hysteresis tests. f) Response and relaxation time. g) The working stability test of the sensor loading over 10,000 times at a pressure of 1 kPa. Schematic diagrams showing the test platform and the normalized change in capacitance as a function of pressure for the sensor under different mechanical deformation. h) The test of the sensor under bending is realized on a cylindrical surface. i) The test of the sensor under bending and stretching is realized on a spherical surface.

The capacitive pressure sensor based on embedded LM‐ME was fabricated by assembling the top layer with an LM‐based plate electrode and the bottom layer with embedded LM‐ME (**Figure** **3**a). The specific preparation process is shown in Figure S8 (Supporting Information). The preparation process of the top LM plate electrode layer involves laser pre‐procession of the PDMS substrate to introduce bumps, followed by stenciling a thin layer of LM on its reverse side and encapsulating it with a PDMS film. Subsequently, the sensing unit area of both the top LM plate electrode and bottom LM‐ME layers were sprayed with SiO_2_ nps. Together with the thin film, they serve as the dielectric layer of the sensor and provide isolation between the two electrodes. Finally, the top and bottom layers were bonded together by oxygen plasma to assemble the sensor array. The presence of SiO_2_ nanoparticles contributes to the reduction in viscosity at the contact interface of thin PDMS layers, thereby mitigating hysteresis in sensor response. (Figure S9, Supporting Information). The SEM of the cross‐sectional view of the assembled sensor is shown in the Figure 3b. It can be seen that the upper and lower layers are tightly bonded together without gaps. The thickness of the sensor is 600 µm, and the height of the bump is 200 µm. The area of the sensor array is 33.5 × 33.5 mm^2^, with each sensing pixel having a radius of 1 mm and spacing between pixels set at 3.5 mm; additionally, connecting lines have a width of 0.5 mm (Figure S10, Supporting Information).

The capacitance response to the pressure of the sensor is shown in Figure 3c. The sensitivity of the capacitive pressure sensors is defined as S = δ(ΔC/C_0_)/δP, where ΔC = C−C_0_ is the relative change in capacitance, C and C_0_ are the capacitance of the sensor with and without pressure applied, respectively, and P is the applied pressure. The sensor exhibits a maximum sensitivity of up to 7.42 kPa^−1^ in the pressure range of 0–0.1 kPa due to the existence of fabricated LM‐ME. The sensitivity of the sensor with different heights of the microstructured electrode was evaluated as illustrated in Figure S11 (Supporting Information). It was observed that with the increase in the height of the microstructured electrode, the sensitivity of the sensor increases accordingly. This result can be attributed to the lower Young's modulus of higher microstructures that are easier to destabilize and have more space to be compressed. The normalized capacitance change (ΔC/C_0_) of our LM‐ME devices can reach up to ≈2 at 20 kPa, which is 10 times that of the conventional sensor based on microstructured dielectric layers (sensor without LM‐ME: ΔC/C_0_ ≈0.25; black circles). To elucidate the pressure sensing mechanism, we simplified the capacitor model and extracted the deformed configurations of the microstructured interfaces (Note S1 and Figure S12, Supporting Information). Compared with the conventional sensor based on microstructured dielectric layers, it results in better‐sensing properties. The soft pressure sensor demonstrated higher performance than other LM‐based pressure sensors reported in the literature (Table S1 and Figure S13, Supporting Information). The real‐time response of e‐skin under different applied pressures from 0.05 to 10 kPa is shown in Figure S14 (Supporting Information). This indicates that the sensor has good stability for pressure response. With such high sensitivity, the device can respond to lightweight substances such as a piece of paper of 0.084 g (the area of paper is 1 × 1 cm^2^, ≈0.84 Pa, as shown in Figure 3d). In addition, the hysteresis of the sensor during one continuous testing cycle is quite small (≈3.6%) (Figure 3e). The response time is another crucial parameter for evaluating the dynamic response of a pressure sensor. We tested the response and relaxation time of our sensor by applying, holding, and removing a pressure of 1 kPa, respectively. Both the response time and the relaxation time were 54 ms (Figure 3f). Mechanical durability is of great importance to the application of flexible pressure sensors. Repeated dynamic compression‐release under a pressure of 1 kPa over 10,000 cycles were conducted, and the sensor showed no significant baseline drift or fluctuation throughout the cyclic test (Figure 3g). The flexible sensor is inevitably deformed in practical application, so it is of great importance to maintain good pressure perception performances under various complex deformations. Due to the unique structure of the sensor, the increase in sensor capacitance caused by strain (0–30%) is much smaller than its response signal under pressure (> 10 kPa). The ratio of ΔC/C_0pressure_ to ΔC/C_0strain_ is within the range of 15% (Figure S15, Supporting Information). The capacitance response of the sensor under pressure was tested at strain levels ranging from 0 to 50%, revealing a decrease in the normalized change in capacitance with increasing stretch rate (Figure S16, Supporting Information). The reason for this is that when the sensor undergoes stretching, there is an increase in the effective area and a decrease in electrode distance, resulting in significant alterations to the quantitative pressure sensing performance. The capacitance responses to pressures of the sensor under bending were also examined (Figure 3h). It was observed that the capacitance change value increased slightly as the bending curvature radius decreased. This phenomenon can be ascribed to the normal pressure exerted during bending, resulting in an overall upward shift of the curve. Furthermore, we conducted experiments to assess the pressure response capability of the sensor on spherical surfaces with varying curvature radius (Figure 3i). During these tests, the sensor underwent a combination of bending and stretching. The results indicate that as the curvature radius of spheres decreases, there is only a slight reduction in the capacitance change. The pressure response of the sensor remains stable within a specific range of deformation and facilitates further expansion to the sensor array for pressure mapping on curved surfaces. This demonstrates the stretchability of the pressure sensor and it still possesses the ability of pressure detecting under mechanical deformations. We conducted compression fatigue tests on the sensor under extreme conditions, including large strain (50%) and high temperature (100 °C) as well as immersing in a chemical solution (acid, alkali, salt), as shown in Figures S17 and S18 (Supporting Information). The experiments have demonstrated the exceptional mechanical, chemical, and physical stability of the sensor, enduring over 10000 cyclic fatigue tests in these challenging conditions. Notably, all these experiments were performed using the same sensor. Due to the melting point of the liquid metal utilized in our experiment being 10.5 °C, experimenting with low temperatures is unfeasible. Nevertheless, this preparation method can be extended to other liquid metals with lower melting points. These excellent performances demonstrated the application potential of the LM‐ME‐based pressure sensor in the field of human wearable electronics and intelligent robotics.

### 2.4 Mechanical Stability and Cross‐Talk Suppression of the Support Isolation Structure

**Figure 4:**
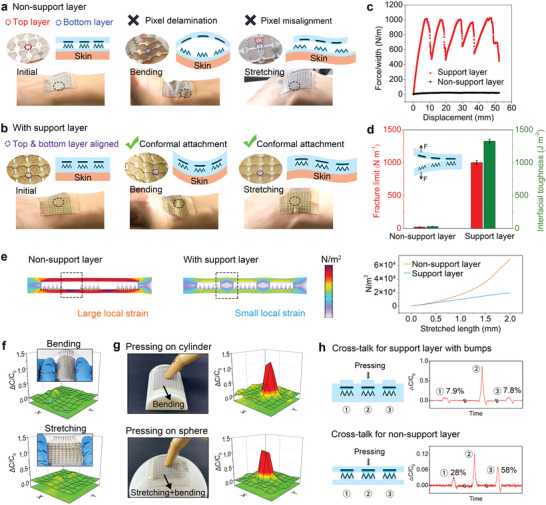
Mechanical and electrical stability testing of the embedded LM‐ME sensor array with support layer. a,b) Interface stability of sensor array with or without support layer when the sensor is attached to a wrist during motion. c) Peel force as a function of displacement for the two configurations. d) Interfacial toughness and fracture limit of the two configurations. e) Stress distribution of simulation results for sensor array with and without support layer under tension displacement from 0 to 2 mm. The support layer enables the small local strain compared with the latter. f) Digital photos and capacitance response of the sensor array under bending and stretching. g) Digital photos and capacitance response of the sensor array under applied pressure on cylindrical (sensor array with bending) and spherical (sensor array with bending and stretching) surfaces. The sensor array has good stability under various mechanical deformations and on different curved surfaces. h) Cross‐talk suppression of support layer and bumps.

The support layer between embedded sensor units can effectively isolate tensile strain and improve the mechanical stability of the sensor array. For comparison, the sensor arrays with or without a support layer are respectively attached to the wrist of the volunteer and constantly move along with the wrist (**Figure** **4**a,b). As for the non‐support layer sensor array, the layers are separated when the wrist is bent upward. When the wrist bends downward (with both bending and stretching for the sensor), the pixels of the upper and lower electrodes are misaligned due to the strain stratification of layers. Significantly, the bonded structure between the top and bottom layer largely avoids the above problems of layer detachment and pixel misalignment. No matter how the wrist moves, the top and bottom electrode layers can adhere tightly together and achieve conformal attachment with human skin. We illustrate the effect of the support isolation structure by comparing the interfacial toughness and fracture limit (or debonding resistance) of the two structures. We conducted a 180° peeling test to measure the debonding resistance and toughness of the four structures, and the results are shown in Figure 4c,d. The non‐support layer is widely used in pressure sensors because it provides a subtly changed interface and thus high sensitivity, but the device is mechanically poor (debonding resistance of ≈0 and interfacial toughness of ≈0). We find that the introduction of the support layer leads to a high fracture resistance (1002 N m^−1^) and high toughness (1328 J m^−2^) between interfaces of the sensor array. It can be seen that the support isolation structure has a toughening and strengthening effect that helps improve the mechanical stability of the sensor array.

Figure 4e shows the simulation results of finite element analysis (FEA) and the stress distributions of the sensor array with a support layer during the gradual tension. The stress is mainly concentrated on the support layer under tension, and the elastic resistance is larger than that of the layered structure without bonded interfaces. The sensor array with a support layer realizes the small local strain, whereas the traditional layered sensor shows the large strain under the same stretched length. Consequently, the adhesive spacers around units increase the energy dissipation and reduce the change of the electrode distance caused by the strain effect (not eliminated) (Figure S19, Supporting Information), thereby reducing the capacitance change during the stretching process as much as possible. Therefore, it can maintain good pressure perception performances under various complex deformations and does not significantly affect the results of pressure mapping of sensor arrays. As shown in Figure 4f,g, we included additional pressure mappings of the sensor under bending conditions as well as on a spherical surface (where both bending and stretching are required) to better align with practical application scenarios in this work. The results demonstrated the excellent stretchability of the sensor array while highlighting its superior performance toward pressure overstrain, thus enabling effective pressure mapping on curved surfaces. The normalized capacitance change (ΔC/C_0_) of bending or stretching does not exceed 0.2, which is negligible compared to ≈13% of the normalized capacitance change (ΔC/C_0_ ≈1.5) under “bending + pressing” or “stretching + pressing” (Figure S20, Supporting Information) or “bending + stretching + pressing” (The pressure of pressing is 20 kPa). The support layer reduces the influence of strain on the pressure sensing signal which would improve the signal‐to‐noise ratio, accuracy, and stability of the pressure sensor array under mechanical deformation.

The support layer and bumps of the sensor array can facilitate the mitigation of signal crosstalk among sensing units. Taking a 3 × 3 array as an example, four kinds of structures are simulated for finite element analysis of structural mechanics (Figure S21, Supporting Information). The common layered sensor without a support layer shares one surface and there are no spacers between the sensing units, resulting in the collapse of the surrounding points when the middle point is pressed. As for the sensor with a support layer, surrounding interlinked spacers can reduce the stress generated by pressing a pixel point to disperse the surrounding pixel points. In addition, the bumps on pixels further concentrate the stress on the unit which further reduces the influence of pressing one point on the surrounding eight‐pixel points. The signal cross‐talk is defined as the signal amplitude of the neighboring sensor about the one under loading. By applying pressure on the middle sensing unit, signal changes of three units were collected in turn and calculated to obtain the signal crosstalk between the sensing units with or without support layer and bumps (Figure 4h). The cross‐talk of the sensor array is 58% without the support layer, while the crosstalk can reduce to 19% with the support layer (Figure S22, Supporting Information). Further, under the synergistic effect of the support layer and the bumps, the cross‐talk can be reduced to 7.8%. For practical application in human‐machine interfaces, data are mostly generated on irregular surfaces, such as human skin, biological tissues, and robotic joints. Such surfaces are usually soft, dynamic, or 3D curved. Table S2 (Supporting Information) illustrates the performance comparison with a state‐of‐the‐art flexible pressure sensor array. Although the table includes sensor arrays with superior sensitivity and selectivity, their limited flexibility hinders practical applications where conforming to irregular curved surfaces is essential. Consequently, our sensors possess significant advantages in such scenarios.

### 2.5 Applications of the Pressure Sensor Array

**Figure 5:**
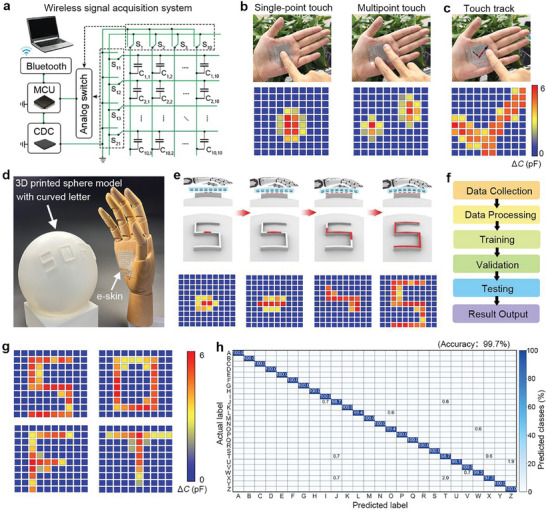
Pressure mapping on curved surfaces with a wireless signal acquisition system. a) Diagram of the wireless signal readout circuit. b) Digital photos and test results of dynamic stimuli including single‐point touch and multipoint touch. c) Digital photos and test results of touch trajectory: drawing a tick and overlapping all signals. d) Digital photos of the sensor array for curved pressure mapping on a 3D printed sphere model. e) Schematic of dynamic tactile recognition process of the sensor array when pressing on the spherical surface with the curved letter “S” and signal mappings. f) Deep learning signal processing flowchart. g) Signal mapping of the pressure sensor array for recognition of curved letters “SOFT”. h) Confusion map of the tested 26 curved letters, showing a recognition accuracy of 99.7%.

A readout circuit is designed to detect the capacitance change of pixels. **Figure** **5**a shows the readout circuit diagram of the wireless signal acquisition system. The sensor array is connected to 10 rows and 10 columns of single‐pole, double‐throw analog switch through a flexible printed circuit connector. The analog switch scans the pixels in the sensor array one by one and shields all other sensing elements to the ground. A cross‐talk compensation module based on the analog switch and the switching circuit was designed to suppress the cross‐talk of the sensing elements in the same row or the same column.^[^ ^4b^ ^]^ A microprogrammed control unit (MCU) that controls all analog switches was used to select the target sensing element and further switched different circuit states through the cross‐talk compensation module. A capacitance‐digital‐converter (CDC) unit is employed to detect changes in capacitance under the corresponding compensation circuit, and the acquired data is processed by the MCU before being transmitted to the host computer via Bluetooth, enabling real‐time visualization on an interface display. The wireless signal acquisition system can monitor real‐time pressure mapping and filter out unwanted noise caused by straining and bending. (Movie S1, Supporting Information).

In practical applications, the surface of the object mainly presents a curved state rather than a flat state. The inherent rigidity of the material and the signal instability caused by the non‐bonded interface, however, limit the use of most flexible sensors in a wide range of tensile and deformation. Our all‐soft sensor array can be attached conformably onto a curved surface and deforms like human skin under pressure, and its high stability enables each sensing unit to operate normally under deformation. We demonstrate that the sensor array is capable of mapping the pressure distribution during either static or dynamic loading on curved surfaces because of robust mechanical stability. Figure 5b shows that the sensor is attached to the palm of a volunteer, and with single‐point and multipoint touch the different positions of the sensor array and record the capacitance changes of all pixels. Soon after that, the signal of the touch trajectory was recorded. By overlapping the signal of each step, the trajectory of the continuous touch can be obtained, such as a pattern of the tick as shown in Figure 5c. The dynamic process of each step is depicted in Figure S23 (Supporting Information). A real‐time visual interface can display the dynamic pressure distribution of the process (Movie S2, Supporting Information).

To further characterize the ability of the sensor array to recognize curved objects, a 3D‐printed spherical resin model with curved letters “SOFT” was utilized for testing (Figure 5d). The dynamic process and recognition results of the curved letters “S” are shown in Figure 5e. The contact area between the sensor and the letter surface is highlighted in red color. Under pressure, the flexible sensor gradually deforms to conform to the curved letter surface, leading to an increase in contact area while detecting dynamic signal distribution throughout this process. Notably, the signal mapping exhibits excellent agreement with distinct regions corresponding to features of the letter, thereby demonstrating that this deformable sensor can effectively operate under deformation conditions while accurately identifying patterns on curved surfaces.

Deep learning has been widely acknowledged for its ability to automatically learn the features of data from pressure sensors.^[^ ^4^, ^22^ ^]^ Here, we collect the array capacitance signals of different curved letters through the circuit. Each letter records 1000 sets of data, among which 700 sets were used for training, 150 sets for validation, and the rest 150 sets for testing. After the data collection, a multi‐layer feedforward neural network of Sigmoid neurons and a softmax output neuron is used for learning (Figure 5f). The visualization results of the pressure mapping of the curved letter “SOFT” are shown in Figure 5g and the 26 curved letters data have distinguishable object features as shown in Figure S24 (Supporting Information). According to the above steps, the classification accuracy rate can reach 99.7% as shown in the confusion diagram of Figure 5h.

It's worth noting that, the sensor array demonstrated its ability in the test to endure continuous compression for a minimum of 26000 cycles. The exceptional performance highlights the outstanding mechanical pressure robustness of the sensor array. Furthermore, we tested the pressure distribution of the letter “S” of the sensor array after multiple stretching cycles (Figure S25, Supporting Information). The experiments demonstrate the excellent mechanical stability of the sensor, as it can withstand 10000 tensile fatigue cycles while maintaining a robust pressure response capability. The sensor exhibits excellent long‐term stability and maintains its robustness and reliability even after continuous tensile strain, thereby confirming its suitability for prolonged usage in practical conditions. This all‐soft pressure sensor array can identify the pattern on the curved surface, which offers great promise in robotic manipulation tasks, wearable devices, and human‐machine interfaces.

## 3 Conclusion

We have reported an all‐soft and embedded‐type 10 × 10 tactile sensors arrays based on the embedded LM‐ME with high sensitivity, stability, and low cross‐talk. By employing a water/hydrogel‐assisted patterning method, the challenge of printing LM on rough surfaces for fabricating embedded LM‐ME is effectively addressed. Consequently, the sensitivity of the capacitance LM‐based pressure sensor is significantly enhanced through an increase in electrode relative area. All soft tactile sensors exhibit a high sensitivity of 7.42 kPa^−1^ in 0–0.1 kPa, low LOD of 0.84 Pa, and a fast response capability of 54 ms and could withstand long‐term pressure cycles. The support layer between embedded sensor units enables the sensor array to maintain pressure sensation under bending and stretching conditions. Combined with the bumps on the surface, the sensor array has low crosstalk performance. These characteristics ensure the sensor's capability to achieve pressure mapping under deformation and deliver high‐quality output in pattern recognition of curved surfaces. The recognition of 26 letters on the curved surface is accomplished by leveraging machine learning techniques, achieving an impressive accuracy rate of 99.7%. This research provides valuable insights for the development and design of all soft high‐performance sensor arrays based on liquid metal.

## 4 Experimental Section

### Materials

The LM (EGaIn) was brought from WOCHANG METAL Co. Ltd, which had a melting point of 12 °C. PDMS substrates were finished thin films purchased from HANGZHOU BALD ADVANCED MATERIALS Co. Ltd. PDMS precursor and curing agent were purchased from Sylgard 184, Dow Corning Co. Ltd. The SiO_2_ nps (20 nm) was purchased from Bohuasi Nanotechnology (Ningbo) Co. Ltd. PET film (10 µm) was purchased from Dongsheng Deyi Adhesive Technology Co. Ltd. Acetone, n‐Hexane (> 98.0%) and PVA (Mw ≈145 000) was purchased from Aladdin Industrial Corporation.

### Fabrication of the Microstructures of the LM‐ME Array and Bumps of the Top Layer

As an extreme manufacturing method, the fs laser could achieve high precision and micro/nanoscale fabrication, and has been used to construct microstructures on the surface of various materials.^[^ ^23^ ^]^ Here, a fs laser beam (with a pulse duration of 50 fs, central wavelength of 800 nm, and repetition frequency of 1 kHz) from a Ti: sapphire laser system (Coherent, Librausp 1K‐he200) was vertically focused onto the surface of the PDMS sheet by a plano‐convex lens (focal length of 200 mm) in air. The PDMS substrate was fixed on a computer‐controlled moveable platform. The laser power was held constant at 400 mW and the moving speed of the platform was 5 mm^−1^s^−1^. As shown in Figure S1 (Supporting Information), the microcones of LM‐ME was structured on PDMS film (300 µm in thickness) by a fs laser orthogonally crossed line‐by‐line ablation, and nanoparticles of surface of capacitive pressure sensor by a fs laser typical line‐by‐line scanning. The pattern template for the array was a homemade light block sheet (Cu sheet processed by a galvanometer processing system), placed between the PDMS film and the plano‐convex lens. Adjusting the AD of laser scanning could realize the structures with different functions needed. The interval of the AD of laser scanning lines was set at 180 µm for the fabrication of the microcone arrays of the LM‐ME and 30 µm for the fabrication of bumps on the surface of the top layer of the sensor array, respectively.

### Assembly of Soft Capacitive Pressure Sensor Array

Two electrode layers with a dielectric layer on the surface were bonded together to integrate as a skin‐like capacitive pressure sensor. The dielectric layer of the sensor was a combination of ultra‐thin PDMS film. The capacitance pressure sensor array consisted of three parts: the embedded LM‐ME, the LM plate electrode, and the middle dielectric layer. The embedded LM‐ME was fabricated as shown in Figure 2c. The LM plate electrode was fabricated by printing using the copper template on the other side of the laser processing bumps structure. The sensing pixel was 2 mm in diameter, the spacing was 3.5 mm, and the width of the connecting line was 0.5 mm. By preparing the diluted PDMS solution, the thickness of the cured film could reach ≈20 µm. Then the PDMS/n‐hexane/curing agent mixture was configured and drop‐coated on the two electrode layers to encapsulate the LM, and the mixture mass ratio was adjusted to 10:1:20 so that the thickness of this layer was ≈20 µm. They were then cured in an oven at 80 °C for 2 h. Subsequently, SiO_2_ nps (0.05 g)/acetone (40 ml) dispersion was sprayed onto the surface to prevent adhesion of the upper and lower cured PDMS thin film. The SiO_2_ nps were only sprayed at the sensing unit, leaving the surrounding bonding area. Finally, the top plate electrode layer and bottom microstructured electrode layers were bonded after oxygen plasma treatment (oxygen flow rate was 15 cm^3^ min^−1^, reaction pressure was 70 Pa, radio frequency power was 90 W, reaction time was 50 s); the assembled sensor array was heated at 80 °C for 30 min to enhance bonding. The PDMS thin film and the SiO_2_ nps together constitute the dielectric layer of the capacitive pressure sensor. Both ends of the electrodes adhered to FPC lines using photo fixative for connection to the test device.

### Characterization and Measurements

The surface microstructure of the samples was observed by a Flex 1000 scanning electron microscope (SEM; Hitachi, Japan). The wettability of liquid EGaIn droplets and water droplets on the sample surface was investigated by a JC2000D contact angle system (Powereach, China). The 3D morphology of the surface of the micro‐pyramid arrays was characterized through a LEXT‐OLS4000 laser confocal microscope (Olympus, Japan). All capacitance signals were measured by a WK4100 LCR meter (Wayne Kerr Electronics, UK) at a test frequency of 1 MHz. A multifunctional tension/compression testing system (Model XLD‐20E, Jingkong Mechanical Testing Co., Ltd.) was employed to exert and record applied force. Tests of the sensors under tensile strain were performed on a homemade uniaxial stretcher.

Further details of the Machine Learning Data may be obtained on the GitHub repository https://github.com/xjtu‐xuhongyu/Letter‐Mapping.

## Conflict of Interest

The authors declare no conflict of interest.

## Author Contributions

H.Y.L and C.J.Z contributed equally to this work. H.Y.L and C.J.Z conceived the idea and designed the research. H.Y.L performed most of the experiments. H.Y.X. designed the signal acquisition system and prepared the circuit. Z.X.L carried out the structural mechanics simulation. H.Y.L., C.J.Z, and C.L. analyzed the sensing properties. Q.Y. analyzed the interfacial performance. L.K. performed SEM observation. Y.Z.M., J.L.Z., and J.L. help with sensor fabrication and measurements. H.Y.L and F.C. drafted the manuscript, and all authors contributed to the writing of the manuscript. Competing interests: The Authors declare that they have no competing interests. Data and materials availability: All data needed to evaluate the conclusions in the paper are present in the paper and/or the Supplementary Materials.

## Supporting information



Supporting Information

Supplemental Movie 1

Supplemental Movie 2

## Data Availability

Research data are not shared.
