# Supplementary material for: Microstructured Liquid Metal‐Based Embedded‐Type Sensor Array for Curved Pressure Mapping
Source: Adv Sci (Weinh). 2024 Nov 25;12(3):2413233. doi: 10.1002/advs.202413233 (PMC11744523; doi:10.1002/advs.202413233)
Supplement: Supplementary file 1 — Supporting Information [file ADVS-12-2413233-s001.docx]

Supporting Information

Microstructured liquid metal-based embedded-type sensor array for curved pressure mapping

Haoyu Li^1^, Chengjun Zhang^2^, Hongyu Xu^1^, Qing Yang^2,^*, Zexiang Luo^1^, Cheng Li^1^, Lin Kai^1^, Yizhao Meng^1^, Jialiang Zhang^1^, Jie Liang^1^, and Feng Chen^1,^*

^1^ State Key Laboratory for Manufacturing System Engineering and Shaanxi Key Laboratory of Photonics Technology for Information, School of Electronic Science and Engineering, Xi’an Jiaotong University, Xi’an, 710049, PR China.

^2^ School of Instrument Science and Technology, Xi’an Jiaotong University, Xi’an, 710049, PR China.

*Email: chenfeng@mail.xjtu.edu.cn (Feng Chen), yangqing@mail.xjtu.edu.cn (Qing Yang).
(These authors contributed equally: Haoyu Li, Chengjun Zhang.)

**This PDF file includes:**

Note1

Figures S1 to S25

Table S1

Table S2

References

**Other Supplementary Materials for this manuscript include the following:**

Movie S1

Movie S2

**Note1. Principle of the LM-ME enhancing the sensitivity of LM based pressure sensor.**

The capacitance of individual unit was calculated by using the simplified electric circuit model. As shown in the Figure S12, there are three regions in a compressed pyramid unit for capacitance calculation, that is, the contact region I, the slope region II, and the blank region III. For the flat contact region and blank region, the parallel plate model can be used directly. For the slope region, integration was used to adopt the parallel plate model on a curved surface. The total capacitance of the pyramid unit can be calculated by summing the capacitance of the three regions. For LM-ME sensor, the capacitance is calculated as follow:

$$C=C_{Ⅰ}+C_{Ⅱ}+C_{Ⅲ}$$

$$C_{Ⅰ}=\frac{A_{Ⅰ}\varepsilon_{1}}{{2d}_{1}}$$

$$C_{Ⅱ}=\frac{\iiint\frac{A_{Ⅱ}\varepsilon_{a}}{d_{2}(x,y,z)}dxdydz\times\frac{A_{Ⅱ}\varepsilon_{1}}{d_{1}}\times\frac{\frac{A_{Ⅱ}}{cos\beta}\varepsilon_{1}}{d_{1}}}{\iiint\frac{A_{Ⅱ}\varepsilon_{a}}{d_{2}(x,y,z)}dxdydz\frac{A_{Ⅱ}\varepsilon_{1}}{d_{1}}+\iiint\frac{A_{Ⅱ}\varepsilon_{a}}{d_{2}(x,y,z)}dxdydz\frac{\frac{A_{Ⅱ}}{cos\beta}\varepsilon_{1}}{d_{1}}+\frac{A_{Ⅱ}\varepsilon_{1}}{d_{1}}\times\frac{\frac{A_{Ⅱ}}{cos\beta}\varepsilon_{1}}{d_{1}}}$$

$$C_{Ⅲ}=\frac{\frac{A_{Ⅲ}\varepsilon_{a}}{d_{2}}\times\frac{A_{Ⅲ}\varepsilon_{1}}{d_{1}}\times\frac{A_{Ⅲ}\varepsilon_{1}}{d_{1}}}{\frac{A_{Ⅲ}\varepsilon_{a}}{d_{2}}\times\frac{A_{Ⅲ}\varepsilon_{1}}{d_{1}}+\frac{A_{Ⅲ}\varepsilon_{a}}{d_{2}}\times\frac{A_{Ⅲ}\varepsilon_{1}}{d_{1}}+\frac{A_{Ⅲ}\varepsilon_{1}}{d_{1}}\times\frac{A_{Ⅲ}\varepsilon_{1}}{d_{1}}}$$

where AI, AII, and AIII are the electrode area of regions I, II, and III, respectively; ε_a_, ε_1_ are the permittivity of air and SiO_2_ mixed PDMS, respectively; d_1_ is the thickness of SiO_2_ mixed PDMS thin film, d_2_ is the thickness of air dielectric at the blank region I, and d_2_(x,y,z) is the thickness of air dielectric at the slope region II; β is the angle between the oblique side and the right angle side of the microcone. In this design, the thinner the elastomer dielectric, the higher the sensitivity and SNR the sensor can reach.

For conventional sensor without LM-ME (parallel plate device), the capacitance of a microcone unit is:

$$C^{'}=C_{Ⅰ}^{'}+C_{Ⅱ}^{'}+C_{Ⅲ}^{'}$$

$$C_{Ⅰ}^{'}=\frac{\frac{A_{Ⅰ}\varepsilon_{0}}{d_{2}}\times\frac{A_{Ⅰ}\varepsilon_{1}}{d_{1}}}{\frac{A_{Ⅰ}\varepsilon_{0}}{d_{2}}+\frac{A_{Ⅰ}\varepsilon_{1}}{d_{1}}}$$

$$C_{Ⅱ}^{'}=\frac{\iiint\frac{A_{Ⅱ}\varepsilon_{1}}{d_{1}(x,y,z)}dxdydz\times\iiint\frac{A_{Ⅱ}\varepsilon_{0}}{d_{2}(x,y,z)}dxdydz}{\iiint\frac{A_{Ⅱ}\varepsilon_{1}}{d_{1}(x,y,z)}dxdydz+\iiint\frac{A_{Ⅱ}\varepsilon_{0}}{d_{2}(x,y,z)}dxdydz}$$

$$C_{Ⅲ}^{'}=\frac{\frac{A_{Ⅲ}\varepsilon_{a}}{d_{2}}\times\frac{A_{ⅢⅠ}\varepsilon_{1}}{d_{1}}}{\frac{A_{Ⅲ}\varepsilon_{a}}{d_{2}}+\frac{A_{Ⅲ}\varepsilon_{1}}{d_{1}}}$$

where ε_0_ is the permittivity of PDMS, d_2_ the thickness of air dielectric at the blank region I in parallel plate device, d_1_ the thickness of PDMS layer at the blank region I in parallel plate device, d_2_(x,y,z) the thickness of air dielectric at the slope region II in parallel plate device, and d_1_(x,y,z) the thickness of PDMS at the slope region II in parallel plate device.

For the micro-structured electrode sensor, the distance between the upper and lower electrodes is the thickness of the dielectric layer after contact, so the capacitance change during the pressing process depends on the size of the contact area of the plate. In addition, the distance between the microstructured electrode and the dielectric layer and the part not in contact with the plate electrode will also decrease. This change also increases C, but it is small because the distance is much larger than the thickness of the dielectric layer, and the air dielectric constant between the top and bottom layers is very low, which makes the sensor more sensitive than the traditional microstructured dielectric layer design. These excellent performances demonstrated the application potential of the soft haptic interfaces in the field of human wearable electronics. Our fabricated soft haptic interfaces shows extraordinarily high performance superior to that of other reported LM-based tactile sensors in the literature (Table S1).^[1-10]^


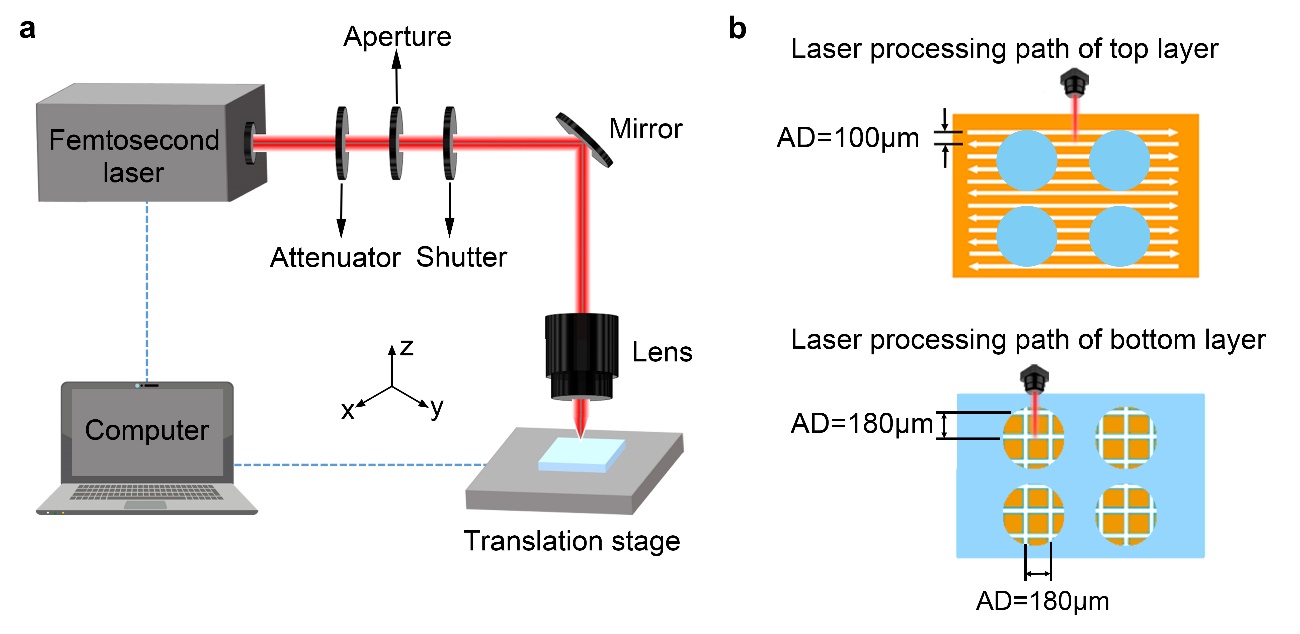


**Figure S1 |** Schematic diagram of femtosecond laser direct writing machining system and the processing path of the top and bottom layers.

**
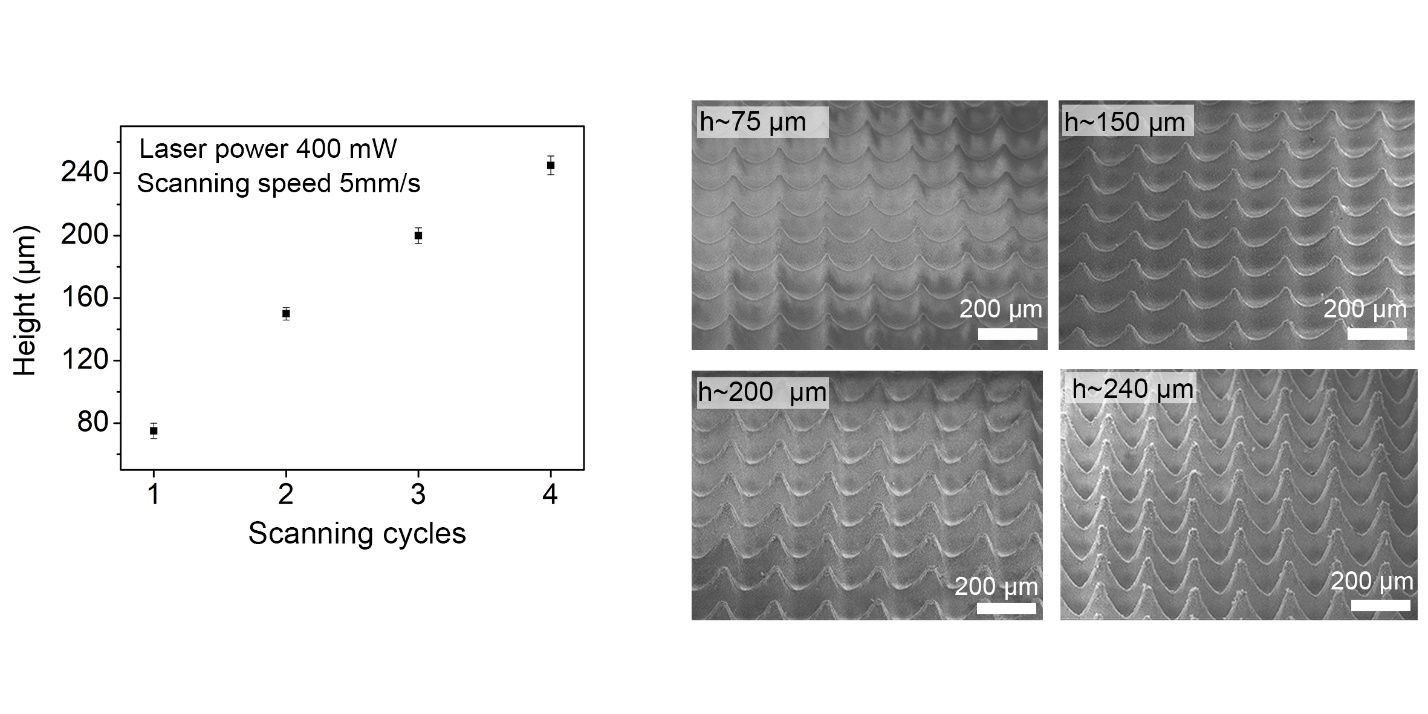
**

**Figure S2 |** The relationship between scanning cycles and height of microcone and SEM images of microcone with different height.


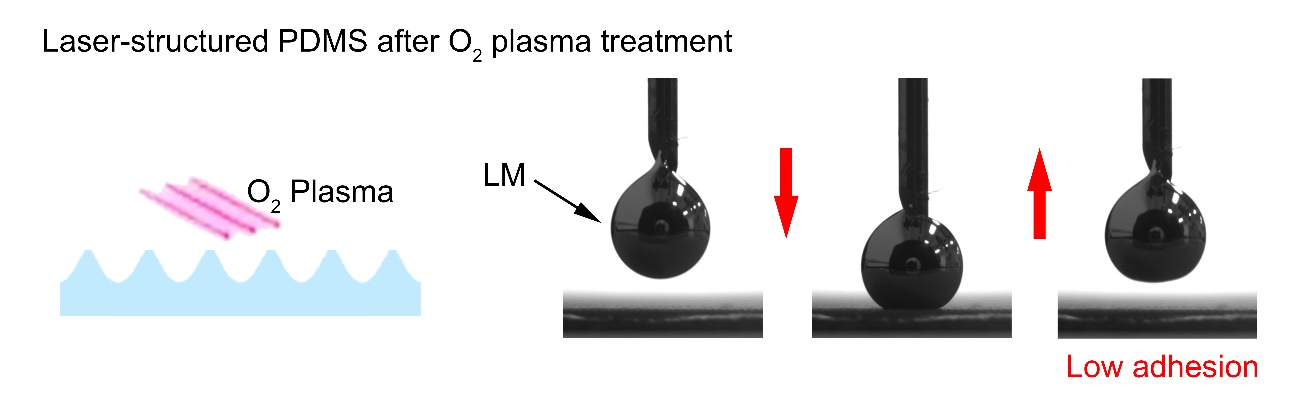


**Figure S3 |** Dynamic wetting behavior of a LM droplet on Fs laser processed PDMS surface after O_2_ plasma treatment (without pre-wetting of water). This low adhesion state indicate that the wettability of LM is not affected by surface chemicals.


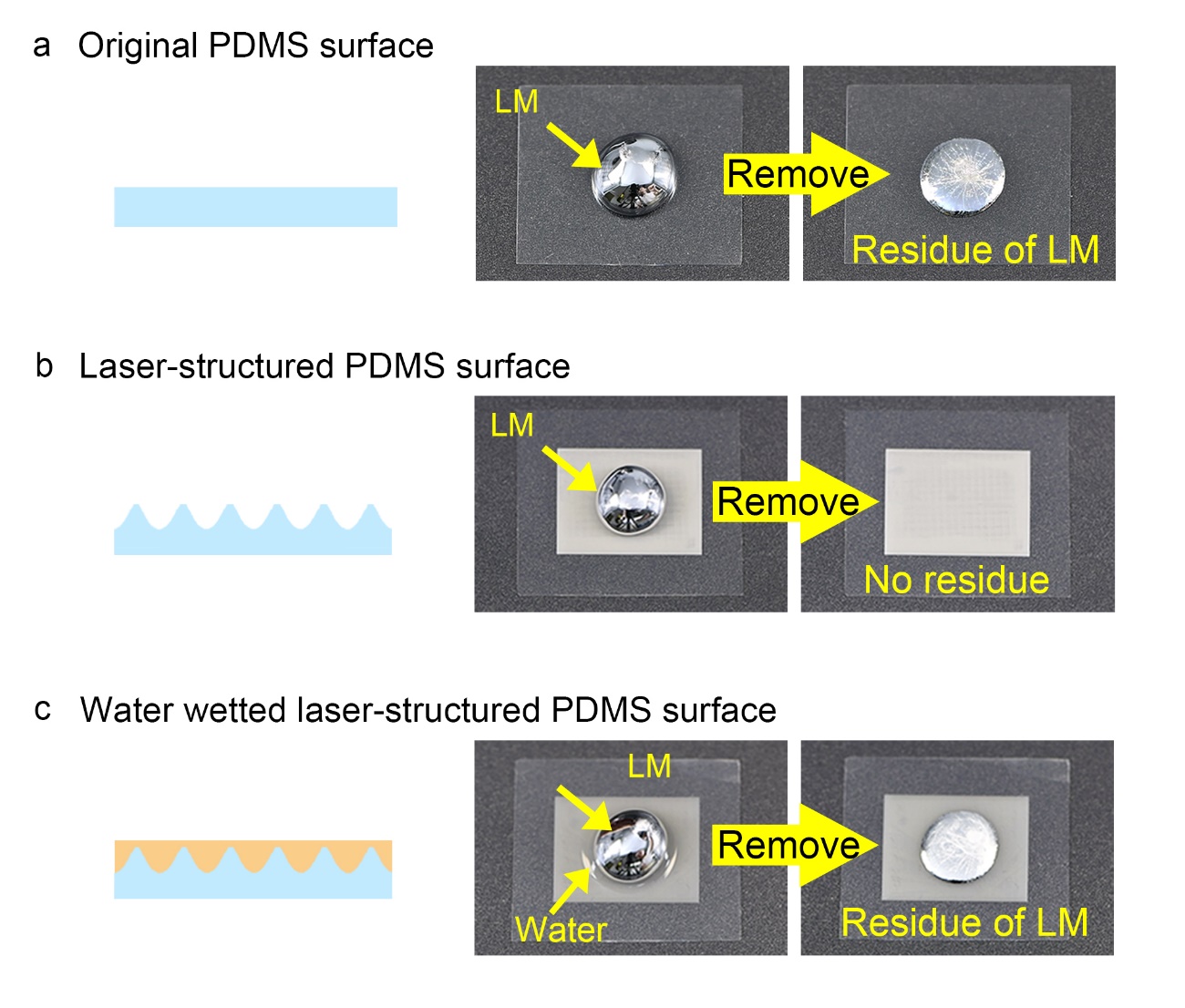


**Figure S4 |** Adhesion test between the LM and the original PDMS surface, the laser-structured surface and the water wetted laser-structured PDMS surface, respectively.


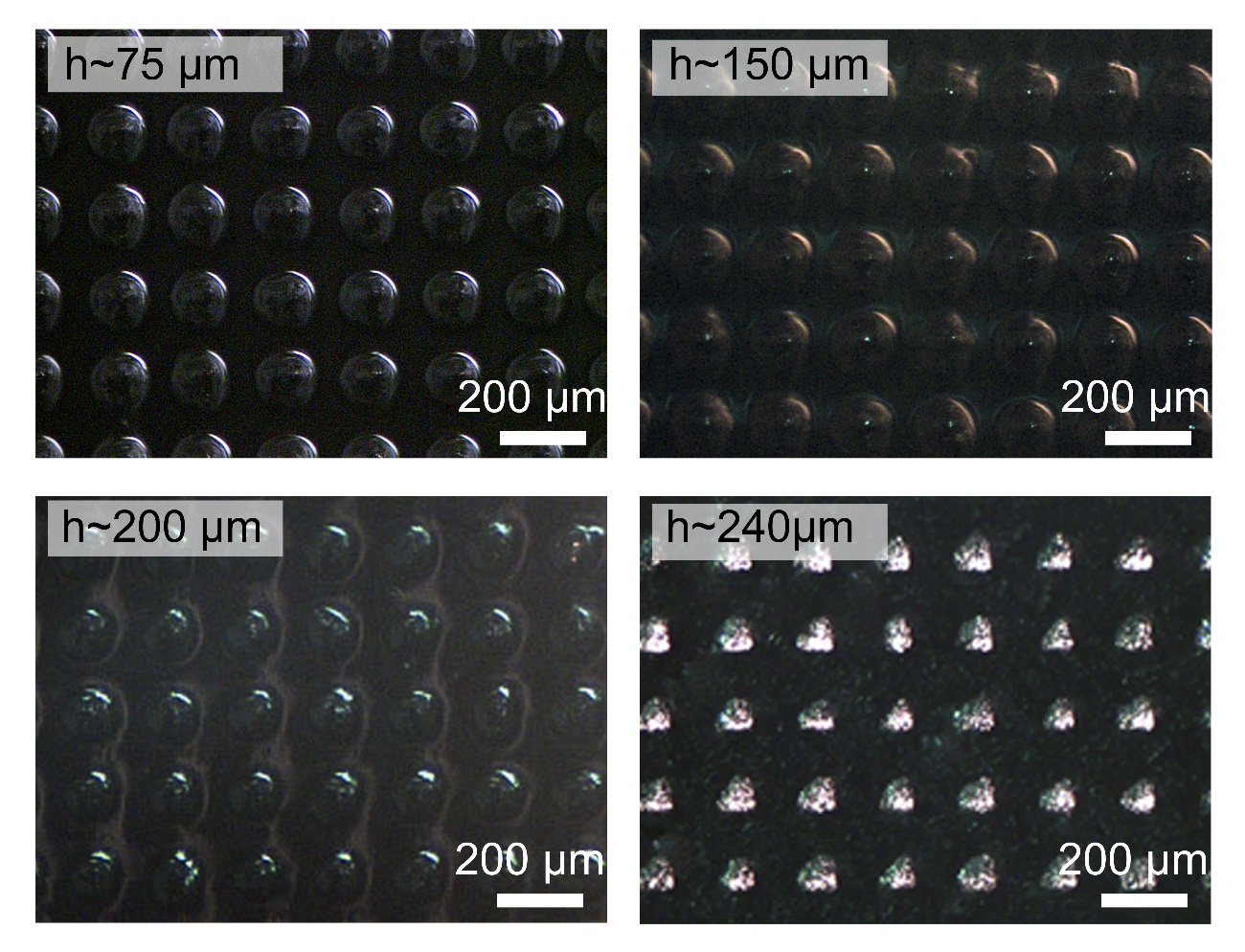


**Figure S5 |** Optical photograph of microcones of different heights covered with LM. When the height of the microcone improved to 240 μm, the LM cannot completely cover the microcones, and the conical tips of PDMS are exposed.


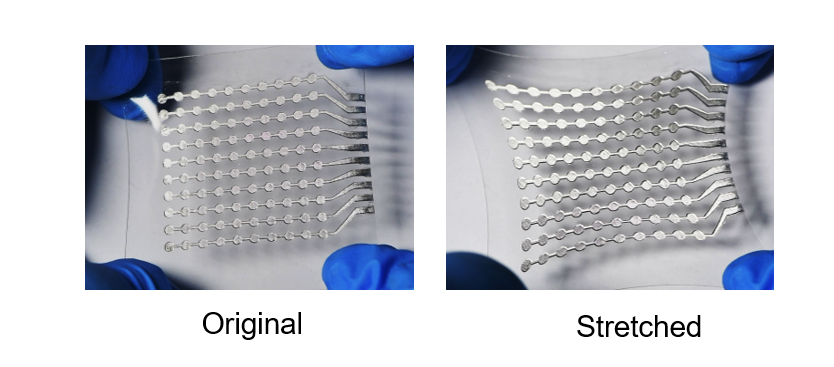


**Figure S6 |**  Digital photo of LM-ME array before and after stretching.


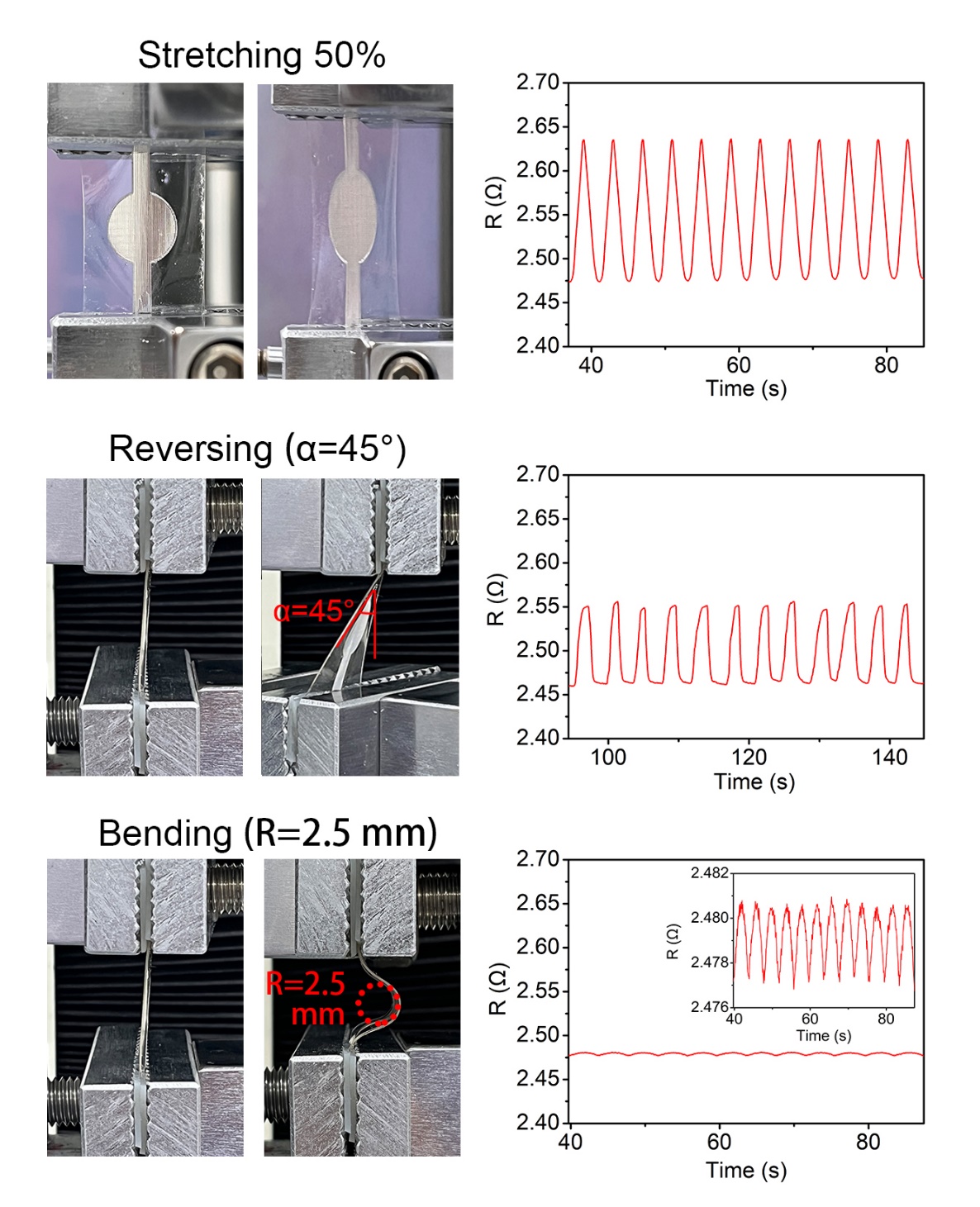


**Figure S7 |** Flexibility and resistance value of microstructured LM electrode under stretching, reversing and bending.


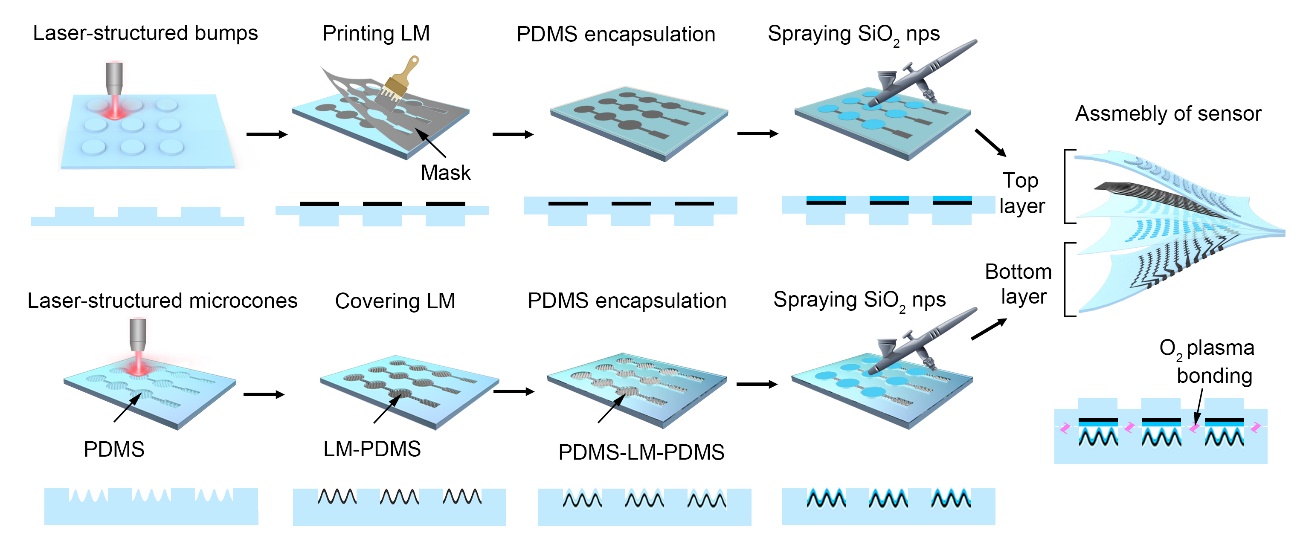


**Figure S8 |** Fabrication of LM based pressure sensor array with embedded senor units.


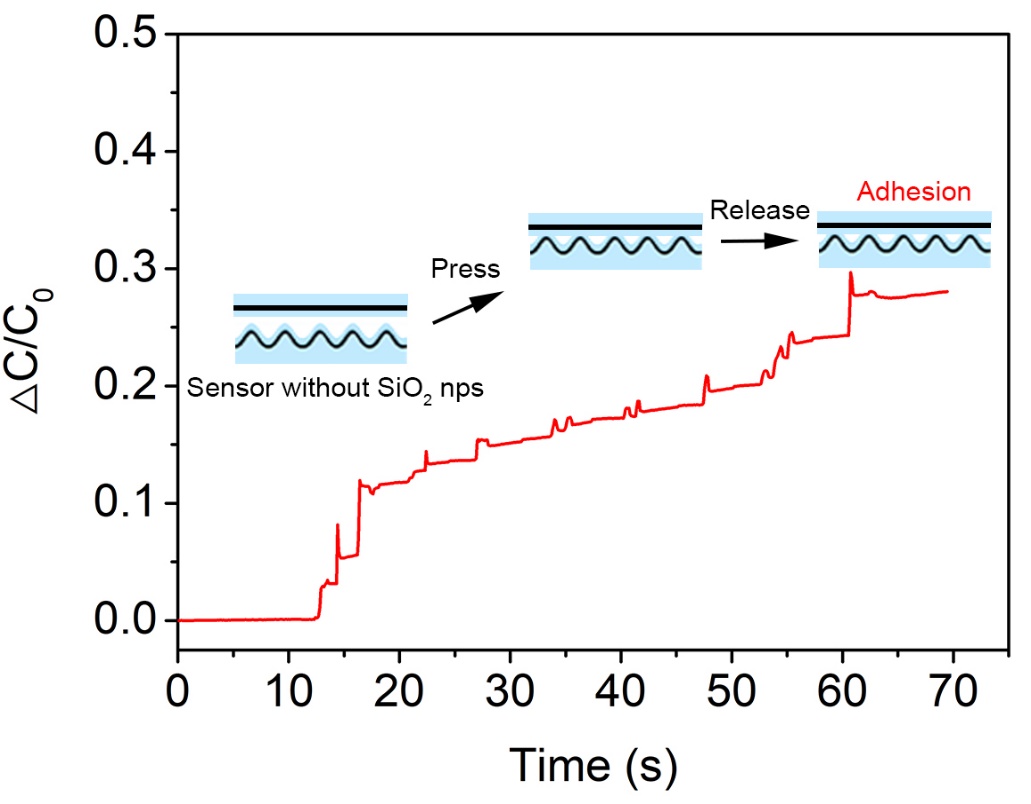


**Figure S9 |**  Sensor without SiO_2_ nanoparticles will adhere under pressure and could not recover initial state after releasing the pressure.


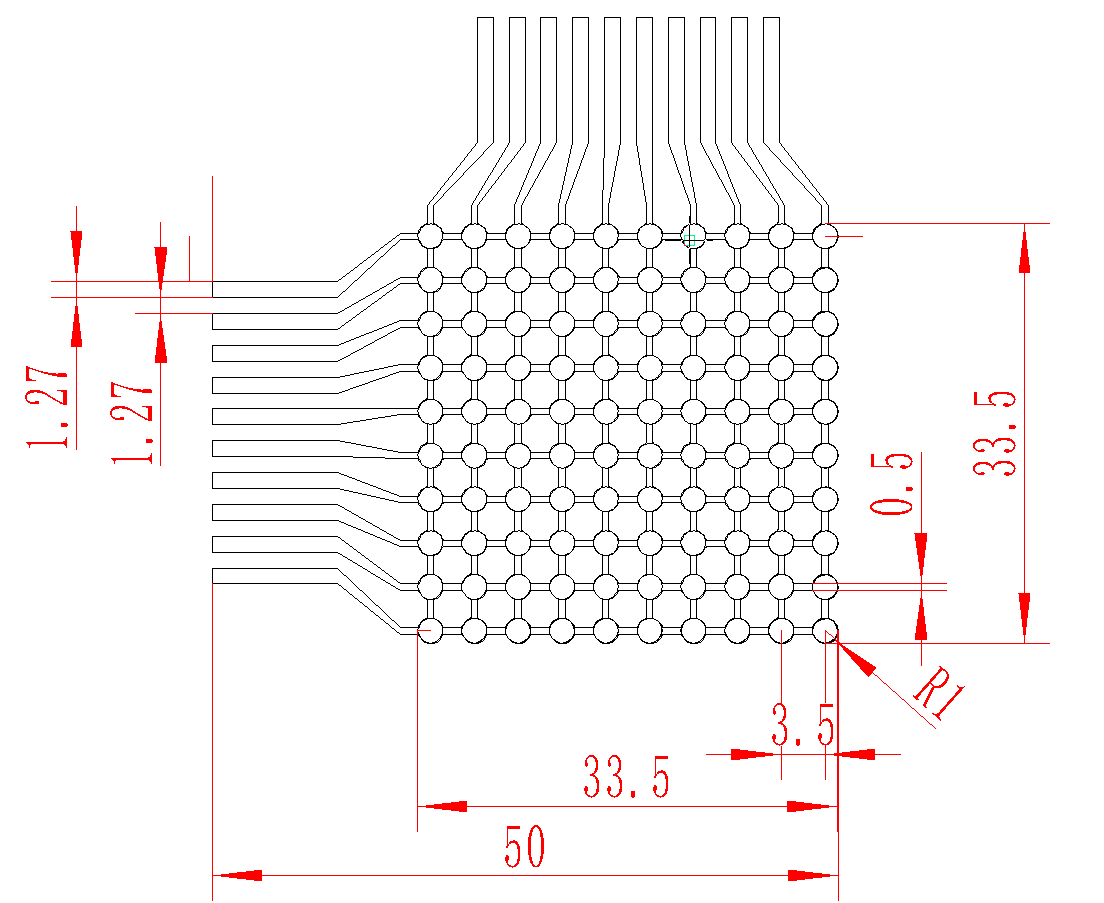


**Figure S10 |** The size of the sensor array. All units are millimeters.


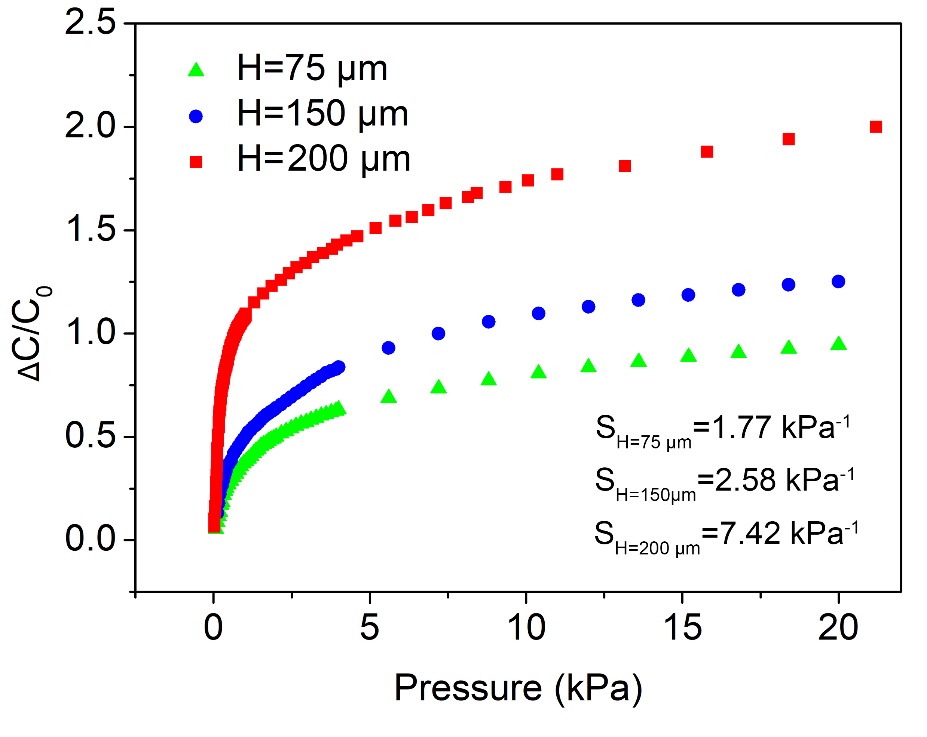


**Figure S11 |** Comparison of the sensitivity of the sensors with different height of microcones.


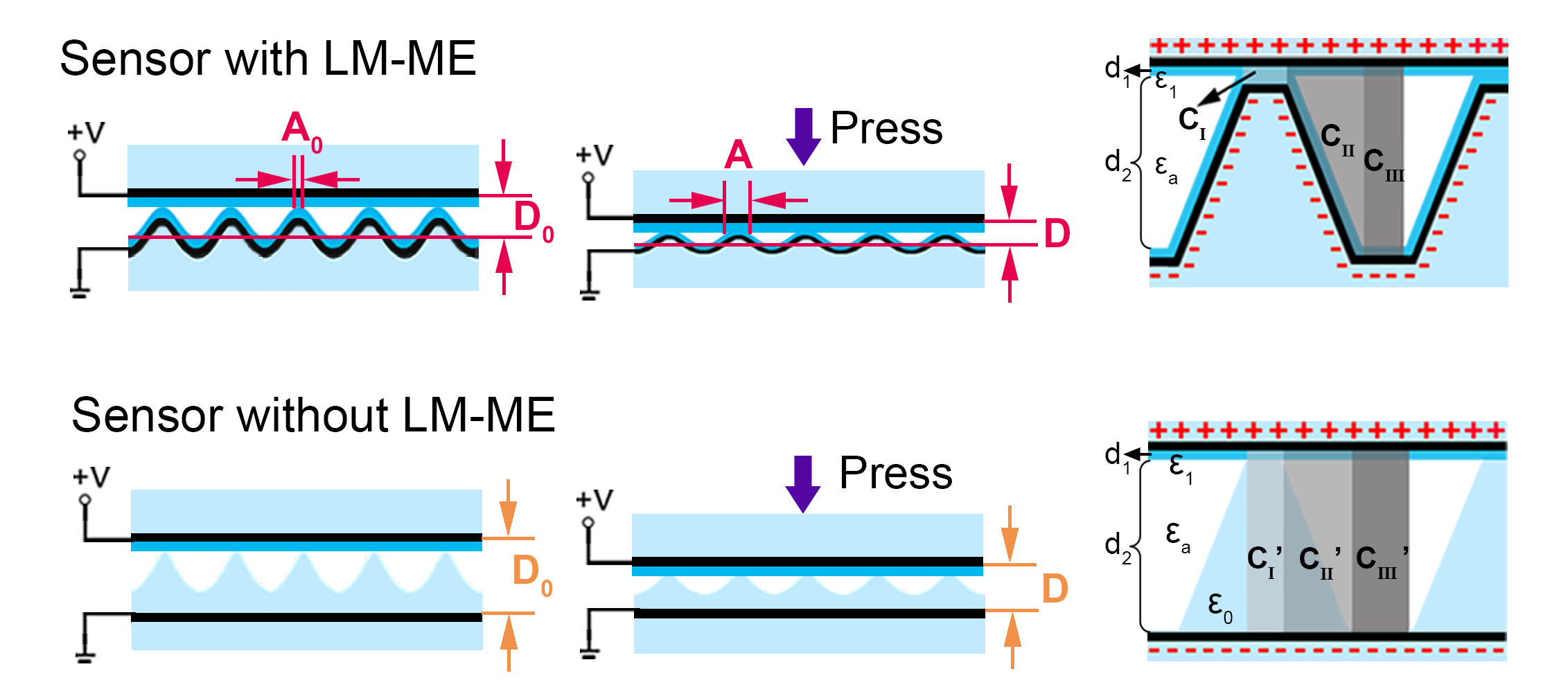


**Figure S12 |** Sensing mechanism for simplified capacitor model of LM-ME sensor and conventional sensor without LM-ME.


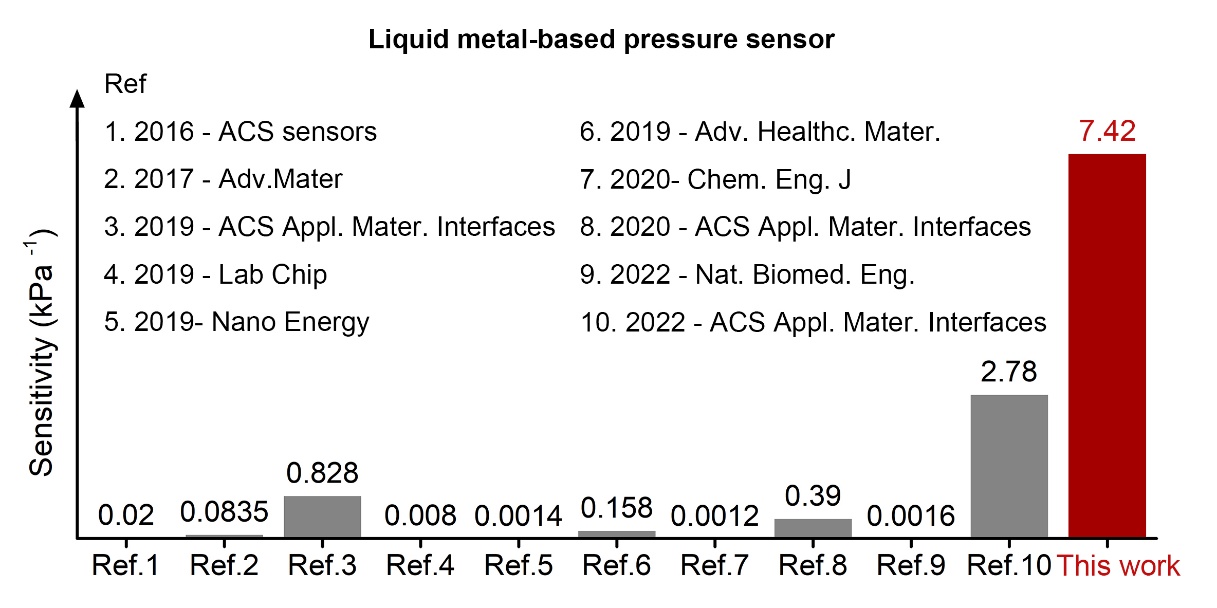


**Figure S13 |** Comparison of the sensitivity of the previously reported LM-based pressure sensor.


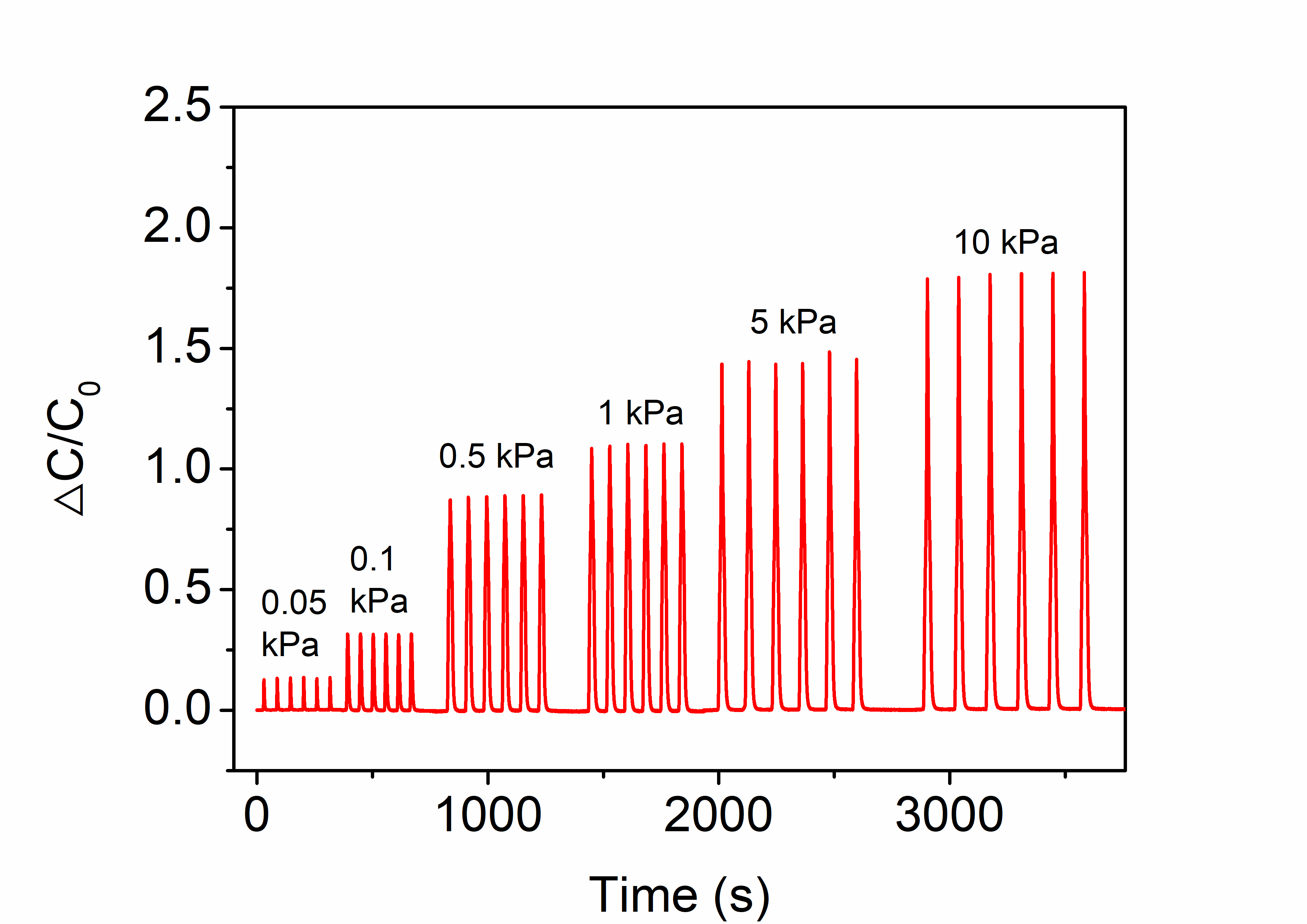


**Figure S14 |** Real-time responses of LM-ME based pressure sensor under different applied pressures.


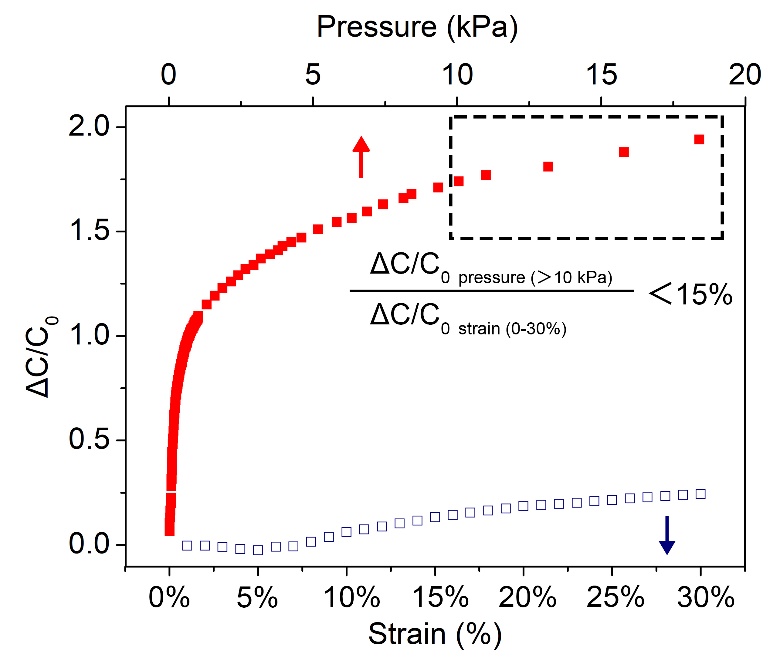


**Figure S15 |** Normalized change in capacitance of the pressure sensor under pressures (up to 20 kPa) and strains (up to strain of 30%).


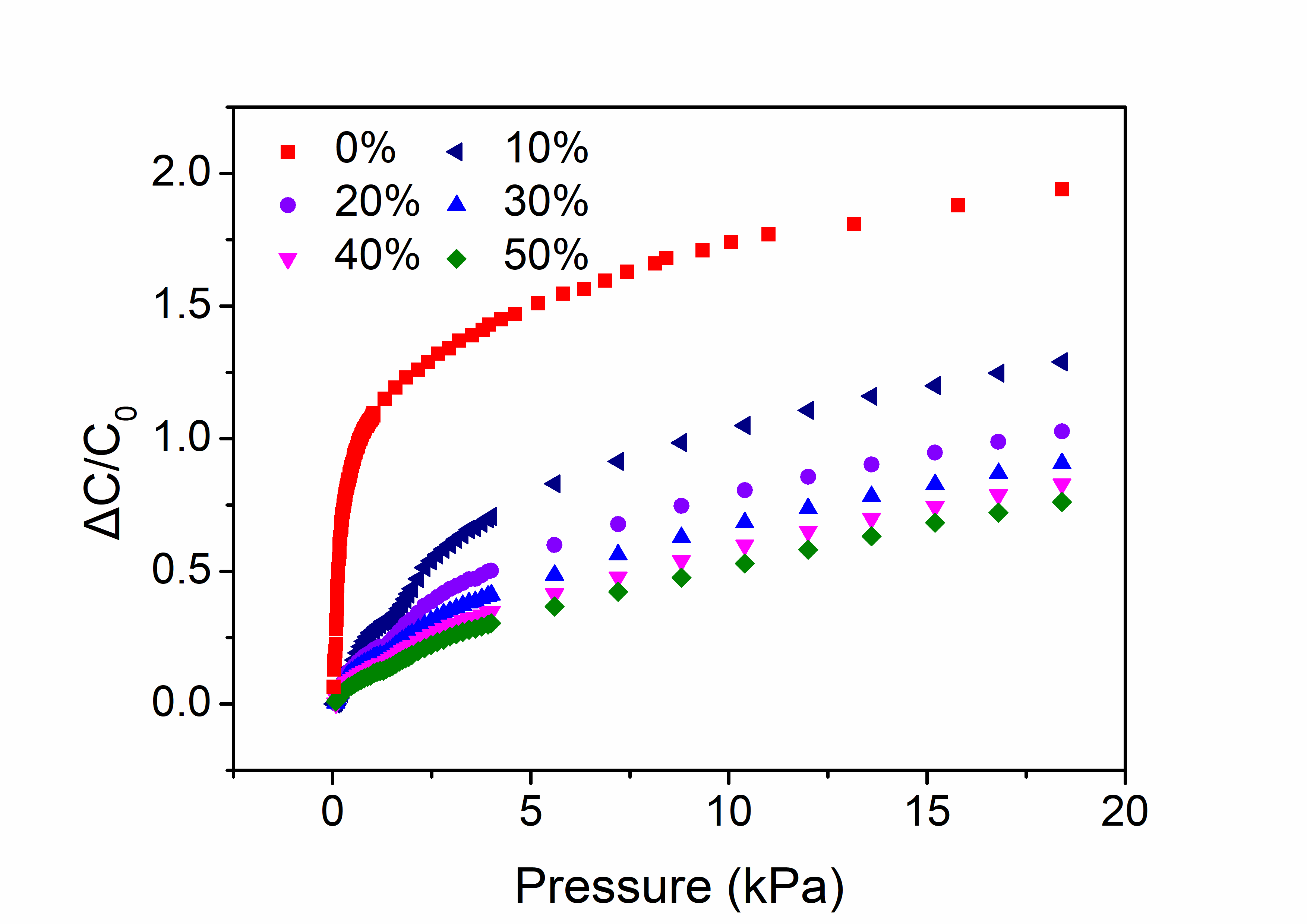


**Figure S16 |** The capacitance responses to pressures of sensor under different strain from 0 to 50%.


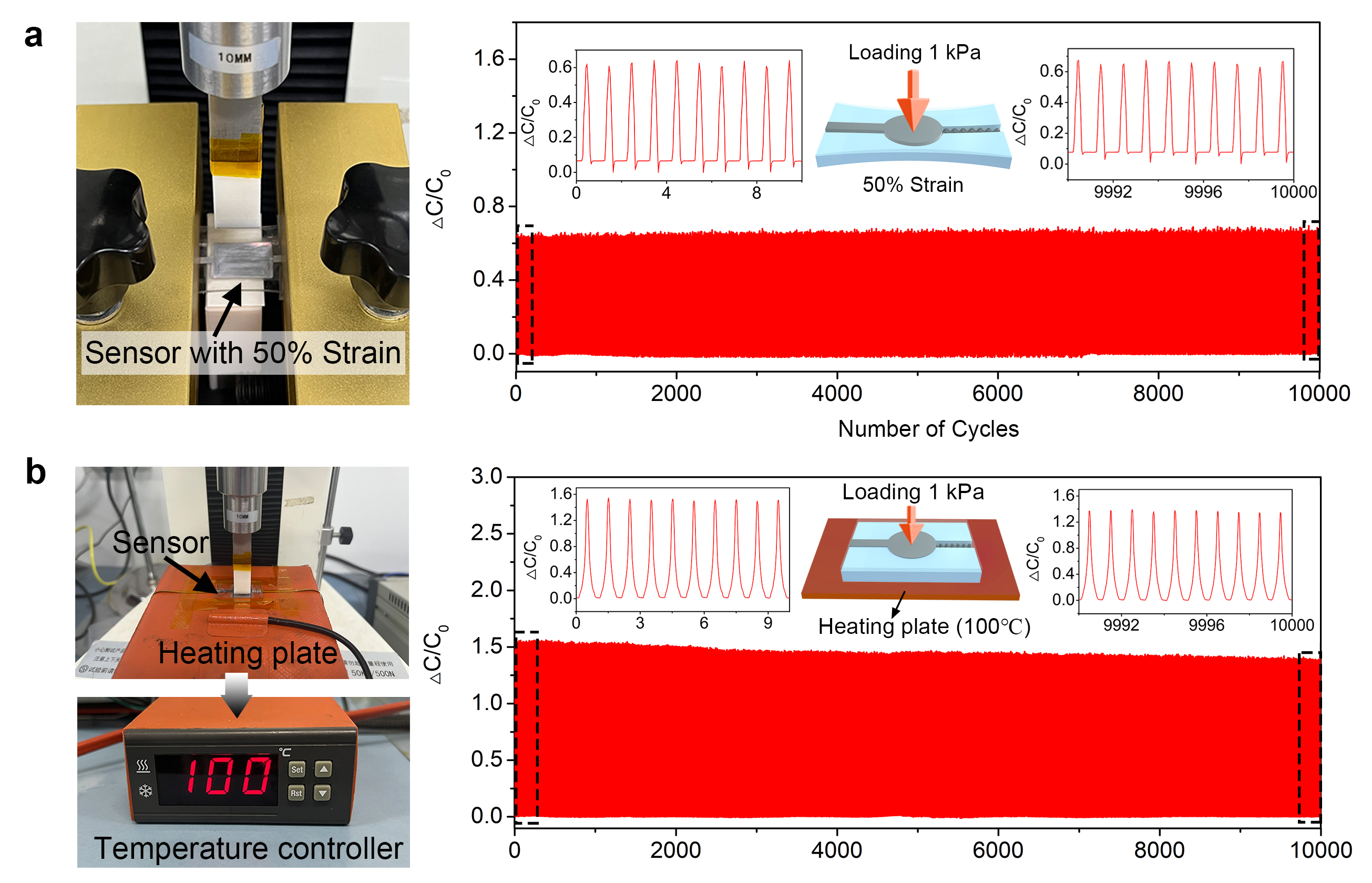


**Figure S17 |** a) Stability of the sensor under repeated pressure (1 kPa) over 10 000 cycles at a temperature of 100℃. b) Stability of the sensor under repeated pressure (1 kPa) over 10 000 cycles at 50% strain condition.


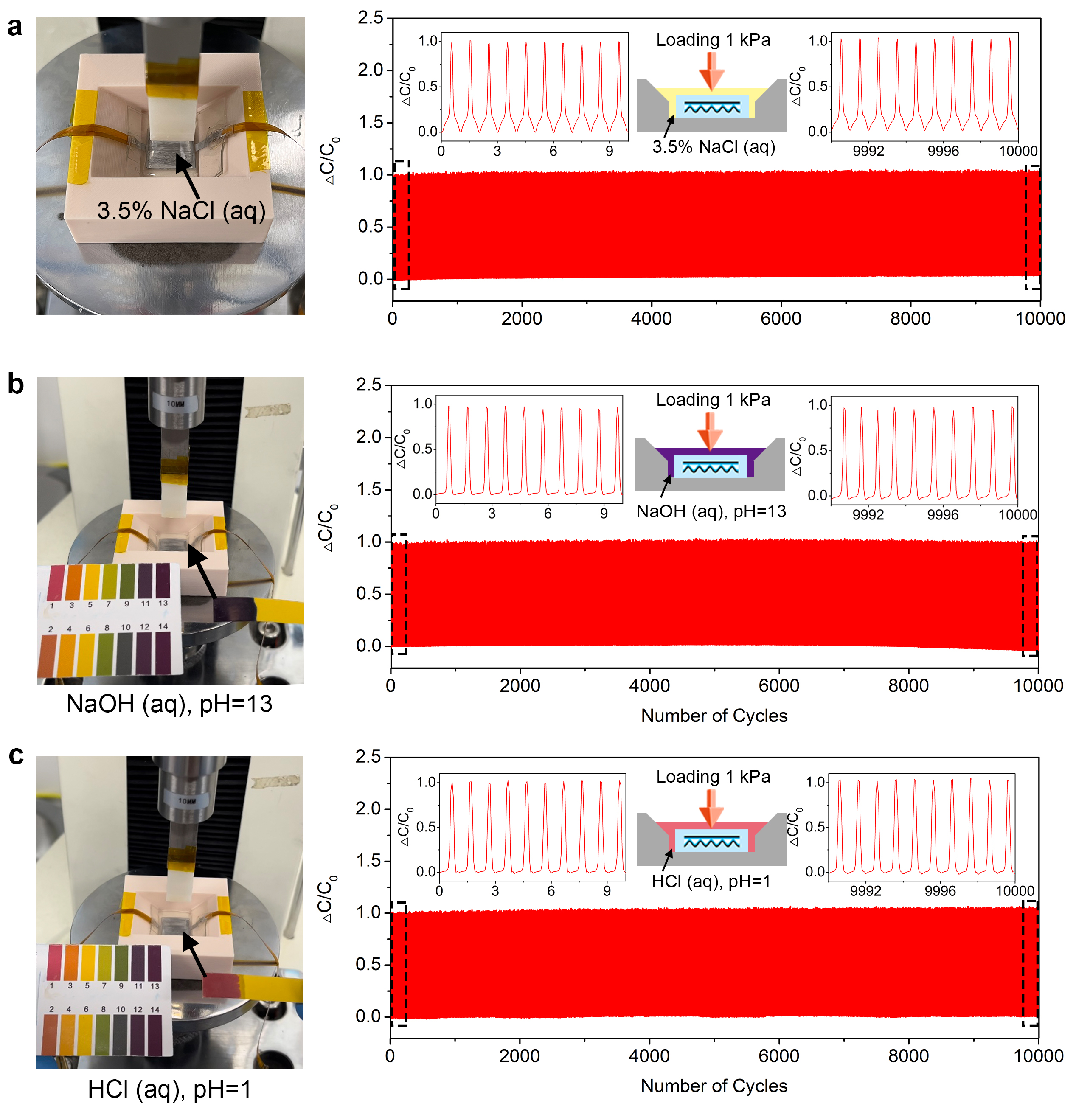


**Figure S18** Stability of the sensor under repeated pressure (1 kPa) over 10 000 cycles while being immersed in a) simulated seawater (3.5% NaCl) solutions, b) NaOH (pH=13) and c) HCl (pH=1), respectively.


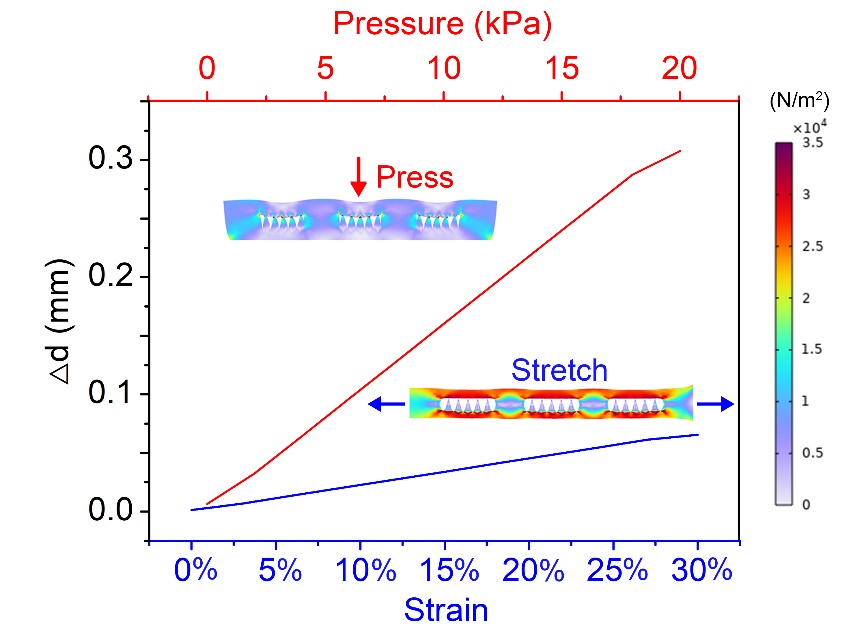


**Figure S19 |** Distance variation between the electrode layers of the isolation interface and stress distribution of simulation results under different strain (0-30%) and pressure range (0–20 kPa).


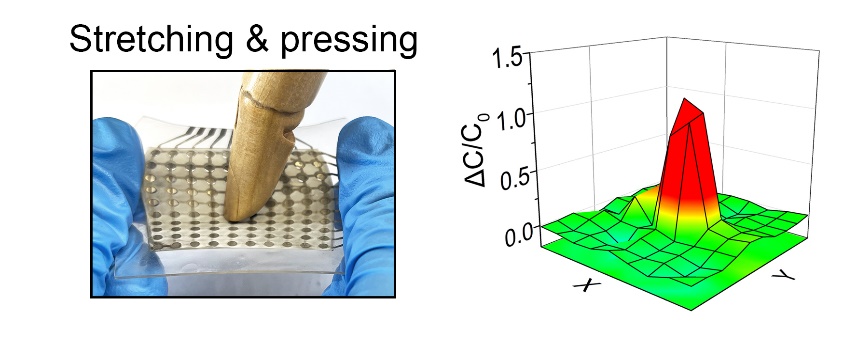


**Figure S20 |** Digital photos and capacitance change of sensor array under stretching and pressing.


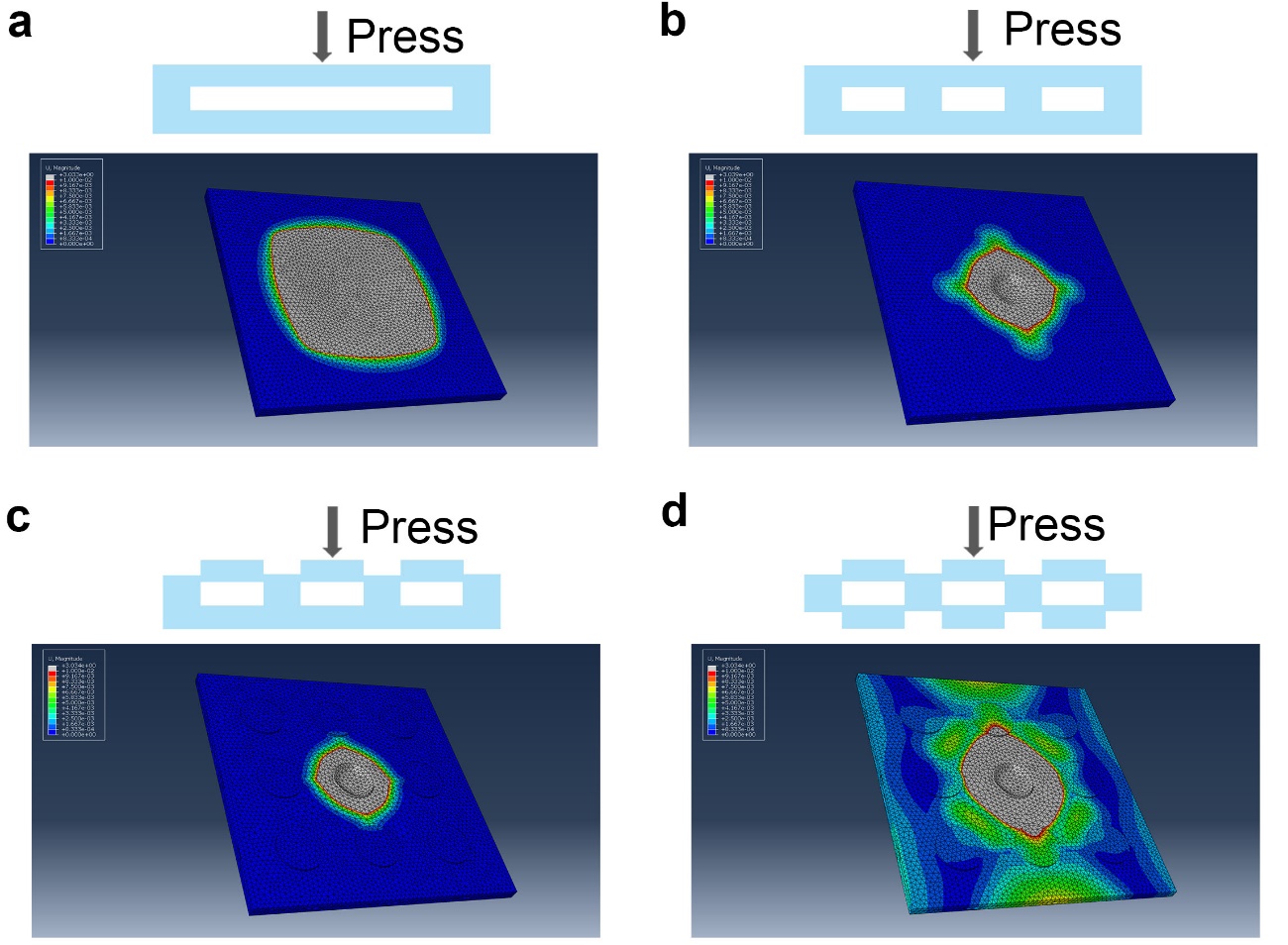


**Figure S21|** Structural mechanics simulation of four structures of sensor array under pressure.


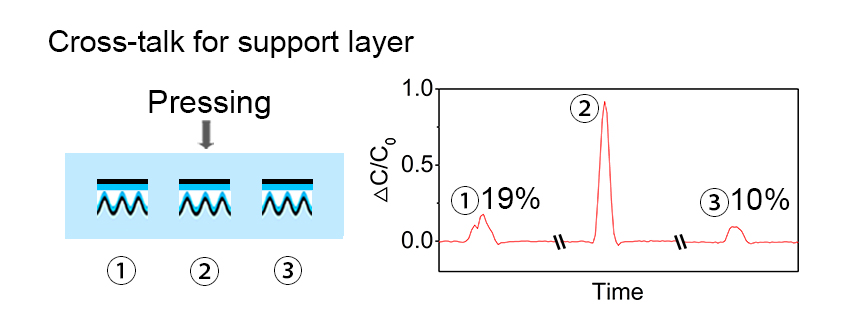


**Figure S22 |** Test of cross-talk of the sensor array with support layer and without bumps on surface.


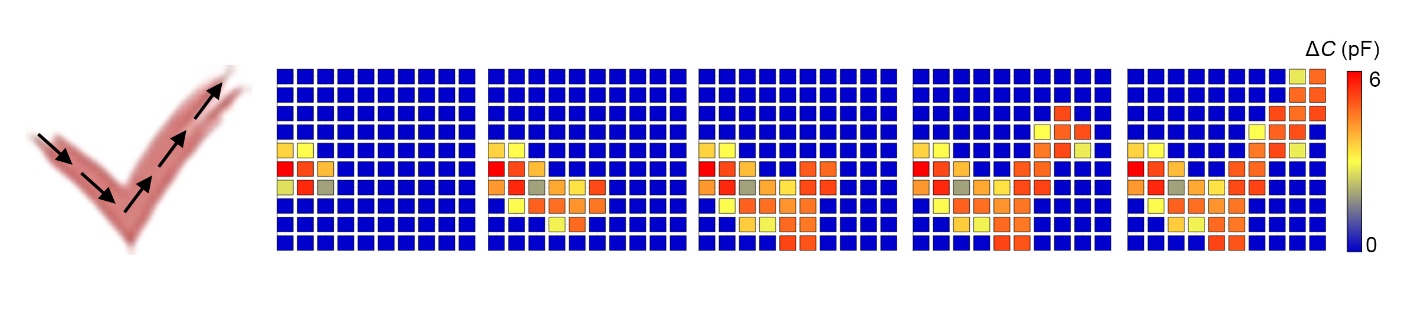


**Figure S23 |** Finger motion track monitoring of drawing a tick by overlapping the signal of each step.

**
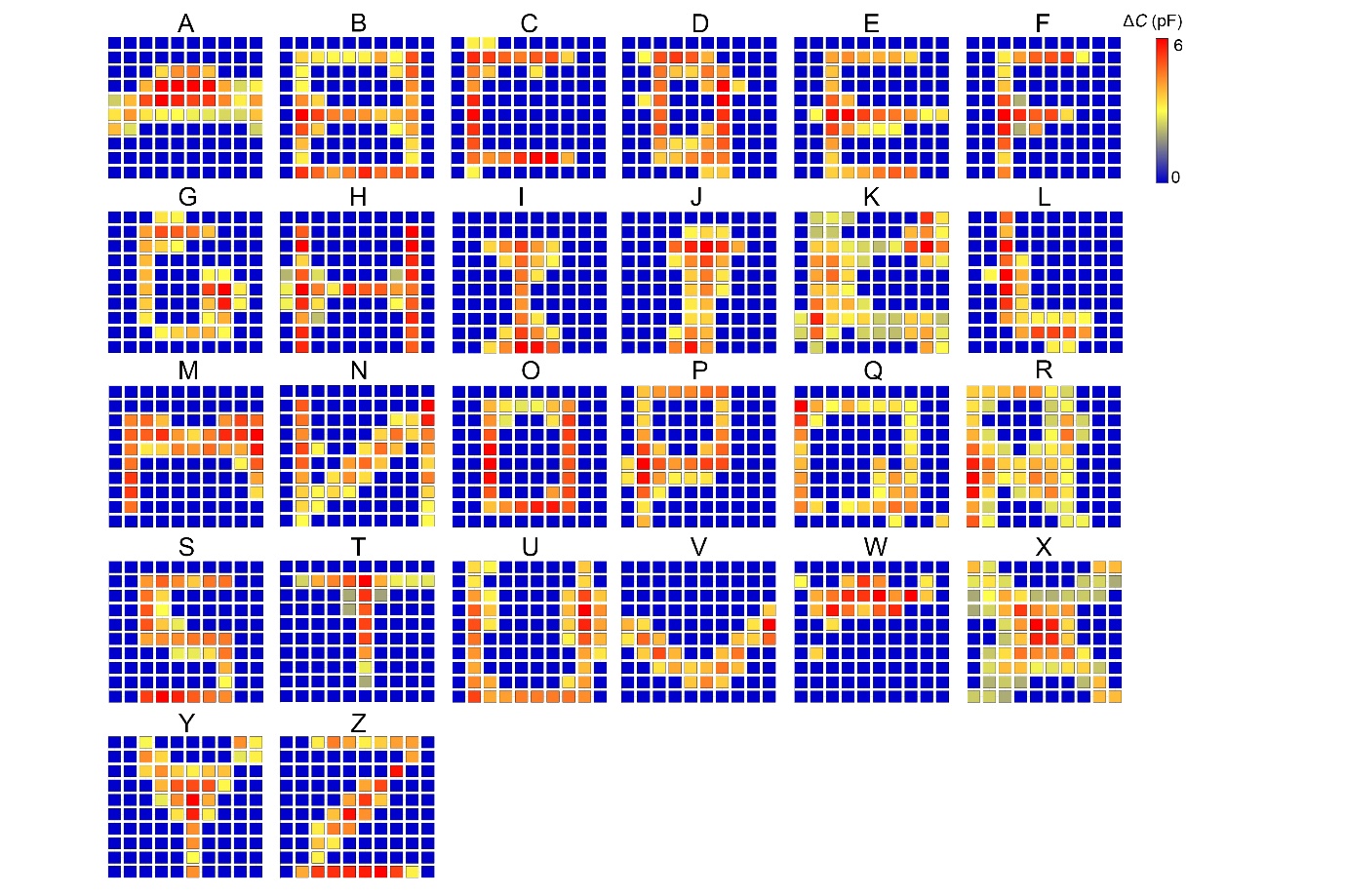
**

**Figure S24 |** Signal mapping for 26 curved letters under pressure.


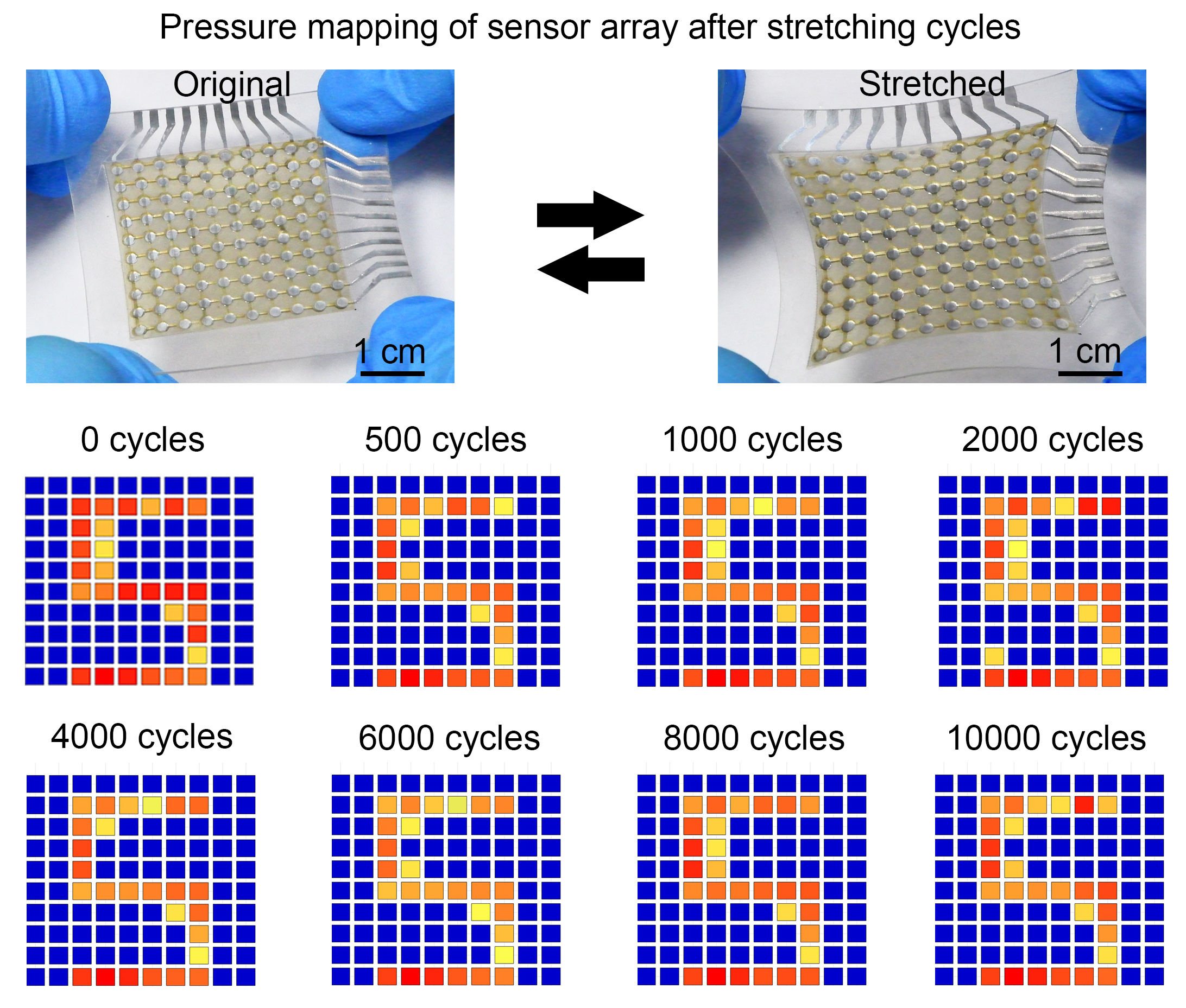


**Figure S25 |** Digital photos of sensor array before and after stretching and pressure mapping of letter “S” by the sensor array after 500, 1000, 2000, 4000, 6000, 8000, 10000 stretching cycles, respectively.

**Table S1** Comparison of the performance of LM-based pressure sensor.

| Structure/materials | Sensitivity /kPa^-1^ | Response time /ms | LOD /Pa | Durability | Number of array | Ref. |
| --- | --- | --- | --- | --- | --- | --- |
| LM-ME | 7.42 | 54 | 0.84 | ＞10000 | 100 | This work |
| Microfluidic diaphragm/LM | 0.0835 | 90 | 98 | / | 1 | ^[2]^ |
| LM-based hydrogel | 0.828 | / | 2000 | ＞480 | 1 | ^[3]^ |
| Microchannel PMDS as dielectric layer/LM | 0.008 | / | 18.6 | / | 100 | ^[4]^ |
| Micro-pillar PDMS as dielectric layer/LM | 0.0014 | 170 | 10 | / | 1 | ^[5]^ |
| Microbump array/LM | 0.158 | 77 | 16 | ＞10000 | 16 | ^[6]^ |
| LM-PVA film | 0.0012 | / | / | ＞1000 | 1 | ^[7]^ |
| Icicle-shaped LM electrodes | 0.39 | 190 | 12 | ＞6000 | 1 | ^[8]^ |
| Qiupu geometry/LM | 0.0016 | 400 | 2000 | ＞500 | 1 | ^[9]^ |
| Double-side micropyramids PDMS as dielectric layer/LM | 2.78 | 80 | 3 | ＞10000 | / | ^[10]^ |

**Table S2** Performance comparison of state-of-the-art flexible pressure sensor array.

| Materials | Sensing mechanisms | Sensitivity /kPa^-1^ | Stretchability/% | Number of array | Crosstalk | Cite |
| --- | --- | --- | --- | --- | --- | --- |
| PDMS/LM | Capacitive | 7.42 | 300 | 100 | 7.8% | This work |
| PDMS/CNT/Au | Capacitive | 810 | 150 | 784 | 0.67% | ^[11]^ |
| PDMS/Cu | Capacitive | 0.22 | ＜3 | 49 | Low | ^[12]^ |
| PU/CNT/PHB-PHV | Capacitive | ~0.1 | ＜3 | 25 | Obvious | ^[13]^ |
| PDMS/Al | Capacitive | ~0.02 | ＜3 | 130 | Low | ^[14]^ |
| PDMS/CNT | Capacitive | 0.00023 | 100 | 64 | Obvious | ^[15]^ |
| PET/PDMS/ITO | Capacitive | 0.55 | ＜3 | 64 | Obvious | ^[16]^ |
| PI/Cu | Resistance | 0.19 | ＜3 | 100 | 47.24 dB | ^[17]^ |
| Parylene/Ni/Ti | Resistance | 0.7 | ＜3 | 16 | Low | ^[18]^ |
| PET/Cu/MXene | Resistance | 99.5 | ＜3 | 81 | Low | ^[19]^ |
| SU-8/Au/MoS_2_ | Resistance | 0.011 | ＜3 | 64 | 24.8 dB | ^[20]^ |
| TPU/PI/Cu | Resistance | 21.5 | 66.7 | 64 | Low | ^[21]^ |
| PDMS/Ag NFs | Resistance | 18.94 | 50 | 36 | 33.41dB (9.7%) | ^[22]^ |

**Movie S1**: Real-time visual display of pressure sensor array under stretching, bending and stretching & pressing.

**Movie S2**: Real-time visual display of pressure sensor array under single-point, multipoint touch and continuous touch.

References

[1] J. C. Yeo, Kenry, J. Yu, K. P. Loh, Z. Wang, C. T. Lim, ACS Sensors 2016, 1, 543.

[2] Y. Gao, H. Ota, E. W. Schaler, K. Chen, A. Zhao, W. Gao, H. M. Fahad, Y. Leng, A. Zheng, F. Xiong, C. Zhang, L. C. Tai, P. Zhao, R. S. Fearing, A. Javey, *Adv Mater* **2017**, 29, 1701985.

[3] M. Liao, H. Liao, J. Ye, P. Wan, L. Zhang, *ACS Appl Mater Interfaces* **2019**, 11, 47358.

[4] X. Zhou, R. Zhang, L. Li, L. Zhang, B. Liu, Z. Deng, L. Wang, L. Gui, *Lab Chip* **2019**, 19, 807.

[5] C. Zhang, S. Liu, X. Huang, W. Guo, Y. Li, H. Wu, *Nano Energy* **2019**, 62, 164.

[6] K. Kim, J. Choi, Y. Jeong, I. Cho, M. Kim, S. Kim, Y. Oh, I. Park, Adv Healthc Mater **2019**, 8, e1900978.

[7] Y. Lou, H. Liu, J. Zhang, *Chemical Engineering Journal* **2020**, 399, 125732.

[8] Y. Zhang, S. Liu, Y. Miao, H. Yang, X. Chen, X. Xiao, Z. Jiang, X. Chen, B. Nie, J. Liu, *ACS Appl Mater Interfaces* **2020**, 12, 27961.

[9] K. Nan, S. Babaee, W. W. Chan, J. L. P. Kuosmanen, V. R. Feig, Y. Luo, S. S. Srinivasan, C. M. Patterson, A. M. Jebran, G. Traverso, *Nat Biomed Eng* **2022**, 6, 1092.

[10] C. Zhang, Z. Li, H. Li, Q. Yang, H. Wang, C. Shan, J. Zhang, X. Hou, F. Chen, *ACS Appl Mater Interfaces* **2022**, 14, 38328.

[11] J. Shi, Y. Dai, Y. Cheng, S. Xie, G. Li, Y. Liu, J. Wang, R. Zhang, N. Bai, M. Cai, Y. Zhang, Y. Zhan, Z. Zhang, C. Yu, C. F. Guo, *Sci Adv* **2023**, 9, eadf8831.

[12] Y. Ji, Y. Zhang, J. Zhu, P. Geng, J. E. Halpert, L. Guo, *Small* **2023**, e2207362.

[13] C. M. Boutry, M. Negre, M. Jorda, O. Vardoulis, A. Chortos, O. Khatib, Z. Bao, *Sci Robot* **2018**, 3, eaau6914.

[14] B. C. K. Tee, A. Chortos, R. R. Dunn, G. Schwartz, E. Eason, Z. Bao, *Advanced Functional Materials* **2014**, 24, 5427.

[15] D. J. Lipomi, M. Vosgueritchian, B. C. Tee, S. L. Hellstrom, J. A. Lee, C. H. Fox, Z. Bao, *Nat Nanotechnol* **2011**, 6, 788.

[16] S. C. Mannsfeld, B. C. Tee, R. M. Stoltenberg, C. V. Chen, S. Barman, B. V. Muir, A. N. Sokolov, C. Reese, Z. Bao, *Nat Mater* **2010**, 9, 859.

[17] H. Luo, X. Chen, S. Li, J. Xu, X. Li, H. Tian, C. Wang, B. Li, M. Zhang, B. Sun, J. He, J. Shao, *Adv Sci (Weinh)* **2024**, 11, e2403645.

[18] G. Y. Bae, J. T. Han, G. Lee, S. Lee, S. W. Kim, S. Park, J. Kwon, S. Jung, K. Cho, *Adv Mater* **2018**, 30, e1803388.

[19] Y. Gao, C. Yan, H. Huang, T. Yang, G. Tian, D. Xiong, N. Chen, X. Chu, S. Zhong, W. Deng, Y. Fang, W. Yang, *Advanced Functional Materials* **2020**, 30, 1909603.

[20] Y. J. Park, B. K. Sharma, S. M. Shinde, M. S. Kim, B. Jang, J. H. Kim, J. H. Ahn, *ACS Nano* **2019**, 13, 3023.

[21] Y. Yuan, H. Xu, W. Zheng, M. Liu, S. Li, J. Yan, D. Wang, K. Liu, H. Zhang, G. Chen, W. Wang, G. Wu, C. Xue, H. Cheng, L. Gao, *Advanced Materials Technologies* **2024**, 9, 2301615.

[22] Y. Zhang, Q. Lu, J. He, Z. Huo, R. Zhou, X. Han, M. Jia, C. Pan, Z. L. Wang, J. Zhai, *Nat Commun* **2023**, 14, 1252.
